# A seeding-based neuronal model of tau aggregation for use in drug discovery

**DOI:** 10.1371/journal.pone.0283941

**Published:** 2023-04-04

**Authors:** Ines S. Amorim, Sylvie Challal, Laetitia Cistarelli, Thierry Dorval, Laurene Abjean, Manuelle Touzard, Nicolas Arbez, Arnaud François, Fany Panayi, Ross Jeggo, Erika Cecon, Atsuro Oishi, Julie Dam, Ralf Jockers, Patricia Machado

**Affiliations:** 1 SERVIER, Institut de Recherches Servier, Croissy-sur-Seine, France; 2 INSERM, CNRS, Institut Cochin, Université Paris Cité, Paris, France; McGill University, CANADA

## Abstract

Intracellular accumulation of tau protein is a hallmark of Alzheimer’s Disease and Progressive Supranuclear Palsy, as well as other neurodegenerative disorders collectively known as tauopathies. Despite our increasing understanding of the mechanisms leading to the initiation and progression of tau pathology, the field still lacks appropriate disease models to facilitate drug discovery. Here, we established a novel and modulatable seeding-based neuronal model of full-length 4R tau accumulation using humanized mouse cortical neurons and seeds from P301S human tau transgenic animals. The model shows specific and consistent formation of intraneuronal insoluble full-length 4R tau inclusions, which are positive for known markers of tau pathology (AT8, PHF-1, MC-1), and creates seeding competent tau. The formation of new inclusions can be prevented by treatment with tau siRNA, providing a robust internal control for use in qualifying the assessment of potential therapeutic candidates aimed at reducing the intracellular pool of tau. In addition, the experimental set up and data analysis techniques used provide consistent results in larger-scale designs that required multiple rounds of independent experiments, making this is a versatile and valuable cellular model for fundamental and early pre-clinical research of tau-targeted therapies.

## Introduction

Tauopathies, including Alzheimer’s Disease (AD), Progressive Supranuclear Palsy (PSP), Pick’s Disease and Corticobasal Degeneration, are a group of neurodegenerative disorders characterized by the abnormal deposition of the microtubule-associated protein tau into filamentous neuronal inclusions. Tau phosphorylation and aggregation are early events that precede neuronal death and functional impairment [1, 2]. Despite their high incidence and severe burden to patients, caregivers, and health systems, the only currently available treatments for tauopathies target disease symptoms, as there are no disease-modifying therapies yet available [2–4].

Nevertheless, tau-targeted therapies are emerging as promising therapeutic strategies for tauopathies such as AD and PSP [5–7]. Tau pathology has been proposed to follow a “prion-like” mechanism, where tau molecules with aberrant conformations (seeds) recruit naïve monomeric tau and promote its templated fibrilization, leading to the propagation of tau pathology throughout the brain [8, 9]. Accordingly, experimental seeding models have been developed in which a cell population is exposed to exogenous tau seeds, which are then internalized by the recipient cells and trigger intracellular aggregation of endogenous tau.

The combination of the nature of tau seeds and the cell types used in *in vitro* tau seeding models is extremely important for the adequate translation of research findings. Common sources of tau seeds include: i) full length or truncated forms of recombinant tau protein, with or without associated mutations, where the use of heparin or other adjuvants catalyses the formation of tau fibrils (PFFs: pre-formed fibrils) [10–13]; ii) mouse brain lysates from aged symptomatic transgenic tau animals, which contain hyperphosphorylated and aggregated tau [14–17]; or iii) post-mortem samples from patients with a variety of tauopathies (PHFs: paired helical filaments) [18–22]. For its ease of use, tau PFFs are the most used source of tau in seeding models, though their relevance to advanced stages of drug discovery is limited. The use of human-derived seeds is the most relevant source of pathological tau, but patient material is not straightforward to obtain and work with. Using brain lysates from tau transgenic animals is a good compromise. As transgenic animals age and tau inclusions become abundant, brain lysates can be used as a source of pathological tau. For instance, when added to HEK cells, lysates from P301S mice promote the formation of insoluble and aggregated tau [15, 16]. *In vivo*, intracerebral injection of those same lysates induces progressive tau pathology in experimental mice [23, 24].

The choice of cell type is equally important when developing a scalable and reproducible model. Immortalized cell lines are easy to handle but they fail to accurately replicate the neuronal environment. hiPSC-derived neurons, particularly the ones derived from patients with tauopathies, are highly relevant in terms of their metabolism and neuronal identity, but, to express 4R tau, require long and sensitive differentiation and maintenance protocols that are not efficient for drug discovery [25]. Rodent primary neurons, on the other hand, are a neuronal population easily available and maintained in most molecular biology laboratories, and with a good potential for clinical translation.

*In vitro* models potentially useful for drug discovery have been developed using cell types such as HEK cells [17, 26], N2a cells [27], rodent primary neurons [26] and hiPSC-derived neurons [25, 28, 29]. They use either over-expression of tau [27] or addition of tau PFFs [17, 25, 28], lysates from transgenic mice [17] or human-derived seeds [26]. However, attempts at combining the use of primary neurons with lysates from transgenic tau models have been limited [30].

In this study, we firstly developed a humanized neuronal model to mimic tau pathology in PSP using sarkosyl-insoluble seeding material from PSP donors. Despite clinically relevant, patient derived material is best suited for small scale studies, due to the difficulty in obtaining seeding-competent tau and to the intrinsically high variability of the seeds obtained. Therefore, we then established and pharmacologically validated a new seeding-based neuronal model of tau pathology using knock-in humanized tau mouse primary neurons and lysates from aged P301S mice. We report that seeded hTau primary neurons were able to create Triton-insoluble tau inclusions with several hallmarks of tau pathology and showed prion-like propagation potency. In addition, we demonstrate that this new neuronal model is easy to use, modulatable and highly reproducible.

## Materials and methods

### Ethics statement

All animal experimental procedures were performed in agreement with the European Communities Council Directive 2010/63/EU and were approved by the Ethical Committee for Animal Experimentation of the Institut de Recherches Servier. Adult mice and pregnant dams were sacrificed by overdose of anaesthetic by inhalation of isoflurane. Mouse embryos (E16) for cell culture experiments were obtained by caesarean section and sacrificed by decapitation.

### Mouse lines

Several transgenic mouse lines were used throughout these studies. All lines were bred and maintained at Taconic Biosciences (Denmark) or Charles River (Lyon, France) and transferred to the Institut de Recherches Servier (Croissy-sur-Seine, France) as needed. Mice were housed under standard environmental conditions (12-hour light-dark cycle, temperature: 22 ± 1°C and humidity: 50%), with *ad libitum* access to food and water.

Heterozygous mice expressing human tau 1N4R with the P301S mutation and WT littermates (referred to as P301S and WT mice, respectively), also known as PS19 mice, were developed by [31]. Mice were obtained from Jackson Laboratories (Stock No: 008169) and bred at Charles River.

Humanized tau knock-in mice (referred to as hTau mice) expressing the human 2N4R tau variant were produced by Taconic Biosciences and maintained on a C57Bl/6 NTac background. Mice were developed by replacing the coding region in the mouse exon 2 and the splice donor-site in intron 2 with the human MAPT equivalent. The human tau protein is expressed under the control of the endogenous mouse MAPT promoter.

Homozygous Tau knock-out mice (referred to as KO mice) were produced by Jackson Laboratories (Stock No: 007251) and maintained on a B6.129X1 background at Taconic Biosciences. Mice were developed by replacing the encoding exon 1 with a neomycin selection cassette via homologous recombination.

### Mouse brain lysates

9 month-old male mice were sacrificed and the cortex rapidly removed and flash-frozen. Frozen tissue was lysed in 100 mg/mL of PBS (-Ca^2+^, -Mg^2+^), using a cooled Precellys® homogeniser. Lysates were sonicated (Q800R2, Sonica) at 4°C for 1 min at 90% power, and centrifuged for 15 min at 21.000g, 4°C. The supernatant was collected, divided in single use aliquots and stored at -80°C. Protein concentration was determined using a Pierce™ BCA Protein Assay Kit (ThermoFisher).

### Homogenous Time Resolved Fluorescence

Homogenous Time Resolved Fluorescence (HTRF, Cisbio) was performed to compare the levels of aggregated tau (Tau aggregation kit, 6FTAUPEG) or total tau (Total tau cellular kit, 64NTAUPEG) across samples. Alpha-tubulin (Alpha-Tubulin Housekeeping Cellular Kit, 64ATUBPEG) was used for normalization of protein levels. Samples were prepared according to the manufacturer’s instructions and fluorescence was read on an EnVision multimode plate reader (PerkinElmer).

### Biosensor assay

The Biosensor Assay uses a double stable HEK293 cell pool expressing LargeBit-K18(P301L) and SmallBit-K18 (S1 File, S1 Fig). Biosensor cells were maintained in DMEM supplemented with 10% FBS, 50 μg/mL Hygromycin B and 5 μg/mL Blasticidin S, in a humidified 37°C, 5% CO_2_ incubator. For analysis of the seeding potency of lysates, cells were plated in poly-D-lysine (100 μg/mL) coated white 96-well plates with optically clear bottom, at a density of 10,000 cells/well. 48h after plating, 100 ng/cell of lysates, sonicated as before, were introduced to cells using Lipofectamine 2000 (ThermoFisher). After 24h, cells were processed for luminescence detection using the Nano-Glo® Live Cell Assay System (Promega) and an EnVision multimode plate reader (PerkinElmer). Each experiment was performed at least 3 times independently. For all experiments, 3–6 wells were used.

### Mouse cortical neuron cultures

Primary neuronal cultures were prepared from hTau E16-17 embryos. Cortices were dissected in Gey’s Balanced Salt Solution supplemented with 1% glucose and digested in 10 mg/mL of papain in DMEM. Cells were plated in poly-D-lysine (100 μg/mL) and laminin (1 μg/mL) coated culture plates at a density of 18,000 or 540,000 cells per well 96- or 6-well plates, respectively. After 4 h in plating media (DMEM, 10% FBS, 1% N2, 2% B-27), media was replaced with complete Neurobasal (Neurobasal, 1% B-27, 1% Glutamax). Cells were kept in a humidified incubator, at 37°C with 5% CO_2_, and half of the media volume was replaced maximum every 7 days.

### Tau seeding in hTau primary neurons

Lentivirus (LVs) were produced by Flash Therapeutics (France). LVs use a human Synapsin I promoter to express human 2N4R tau, fused to a T7 tag at the N-terminal, with (T7-P301S LV) or without (T7-WT LV) a P301S mutation. A control lentivirus (Control LV) with identical backbone but with no additional insertions in the multiple cloning site was used for control experiments.

At DIV4, hTau primary cortical neurons were transduced with LVs at MOI 5. At DIV7, all media containing LVs was removed and mouse or cell lysates, sonicated as before, were added at a concentration of 16–166 pg/cell (multiplicity of seeding, MOS), in a 1:1 mixture of fresh and conditioned complete Neurobasal media. At DIV13-14, 50% of media was replaced. Cells were fixed or lysed at different time-points, specified in the results section or Figure legends. Each experiment was performed at least 3 times independently. For all experiments, 3–6 wells were used.

### Treatment with siRNA and Anle138b

Dharmacon^TM^ Accell^TM^ (Horizon Discovery) siRNA pools were used for knock-down experiments. Stocks of Accell^TM^ Human MAPT (4137) siRNA (hTau siRNA) and Accell^TM^ Non-targeting pool (NTP siRNA) were prepared at 50 μM and working dilutions (62.5 nM– 1 μM) were prepared at 2X in complete Neurobasal media. At the time of treatment, 50% of culture media was replaced with media containing siRNA. Concentrations and time-points for addition of siRNAs are specified in the results section.

Anle138b was solubilized in 10 mM DMSO and added to the neuronal culture at DIV6 and DIV14, at 1–3 μM. Working dilutions were prepared from the DMSO stock in complete Neurobasal media and, at the time of treatment, 50% of culture media was replaced with media containing Anle138b.

Each experiment was performed at least 3 times independently. For all experiments, 3–6 wells were used.

### Cell lysates

hTau primary neurons cultured in 6 well plates were gently washed with ice-cold PBS, scrapped and pelleted by centrifugation. Cell pellets were resuspended in 50 μL of PBS (-Ca^2+^, -Mg^2+^), sonicated as above, and centrifuged for 10 min at 8.000g, 4°C. The supernatant was collected, divided in single use aliquots and stored at -80°C. Protein concentration was determined using a Micro BCA™ Protein Assay Kit.

### Immunofluorescence

For use in immunofluorescence (IF), cells were plated in tissue culture treated black 96-well plates with optically clear bottom (CellCarrier Ultra microplates, PerkinElmer). Cells were washed twice with PBS and fixed for 15 min in 4% PFA and 4% sucrose with or without 1% Triton-X 100 in PBS, depending on the experiment. The presence of 1% Triton-X 100 during the fixation steps removes soluble tau, allowing the detection of tau inclusions from tau overexpression [17, 20]. After fixation, cells were washed with PBS, incubated with 50 mM NH_4_Cl in PBS for 20 min, blocked in 3% BSA in PBS for at least 45 min, permeabilized with 1% Triton-X 100 when needed, and incubated with primary antibodies (Table 1) in 3% BSA for 1 h at room temperature (RT). Cells were extensively washed with 1% BSA and incubated with secondary antibodies (Table 2) and DAPI (Sigma, D8417) in 1% BSA for 1 h. After washes initially in 1% BSA and finally in PBS, cells were stored in PBS at 4°C until imaging.

**Table 1:** Primary antibodies.

Target	Host species	Supplier	Cat. #	Concentration
AT180	Mouse	ThermoFisher	MN1040	1:500
AT8	Mouse	ThermoFisher	MN1020	1:100
MAP2	Chicken	Novusbio	NB300-213	1:4000
MC-1	Mouse	Peter Davies lab		1:500
PHF-1	Mouse	Peter Davies lab		1:500
Tau	Rabbit	DAKO	A0024	1:5000
T7	Rabbit	GeneTex	GTX30555	1:1000
T7	Mouse	Millipore	69522–3	1:1000

**Table 2:** Secondary antibodies.

	Supplier	Cat. #	Concentration
F(ab’)2-Goat anti-Rabbit IgG (H+L) Cross-Adsorbed Secondary Antibody, Alexa Fluor 488	ThermoFisher	A11070	1:1000
F(ab’)2-Goat anti-Mouse IgG (H+L) Cross-Adsorbed Secondary Antibody, Alexa Fluor 568	ThermoFisher	A11019	1:1000
Goat anti-Mouse IgG2b Cross-Adsorbed Secondary Antibody, Alexa Fluor 594	ThermoFisher	A21145	1:1000
Donkey anti-Chicken IgG (H+L), 647H	Interchim	FP-SC1110	1:500
DAPI	Sigma	D8417	1:1000

### Confocal imaging and analysis

3D confocal imaging was performed using a 63x water objective in an Opera Phenix High-Content Screening System (PerkinElmer). Z-stacks containing 3 to 5 planes separated by 1 μm, of 80–120 fields of view, were obtained for each well. Automated image analysis was performed after a maximum intensity projection as a pre-processing using the Harmony 4.9 software (PerkinElmer). “Neuronal area” was defined as the combined area (μm^2^) of MAP2 and T7 signal in each well, which represents the overall area covered by transduced neurons. The “density of AT8 spots” measurement was calculated, for each well, as the number of high intensity spots simultaneously positive for T7 and AT8, normalised to the neuronal area (# spots/μm^2^). Within each individual culture, 3–6 wells were analysed per experimental condition, and the average measurement was used for statistical analyses.

### Statistical analysis

Statistical analysis, described in the figure legends, were performed on GraphPad Prism 7 software. Statistical significance was defined *a priori* for a p-value below 0.05. For all statistical tests, p-values are represented in graphs as: * p<0.05, ** p<0.01, *** p<0.001, **** p<0.0001.

## Results

### Design of a seeding-based neuronal model of tau aggregation

Establishing a biologically relevant neuronal model of tauopathies is an important and challenging matter. To address this issue, we started by developing a model to mimic tau pathology in PSP.

Sarkosyl-insoluble seeding material was prepared from the Globus Pallidus of PSP donors, using an established protocol which has been shown to produced PHFs capable of inducing tau accumulation *in vitro* and *in vivo* [20]. The sarkosyl-insoluble preparations contained aggregated tau, as detected by Tau aggregation HTRF, and, for some PSP donors, induced tau seeding in a HEK293 cell tau Biosensor assay (S2 Fig). Incubation of primary neurons from hTau mice with sarkosyl-insoluble seeding material resulted in the formation of full-length 4R tau inclusions, after thirteen days in culture. The inclusions were positive for conformation dependent MC-1 and AT8 labelling (phosphorylated tau), which are markers of pathological tau species [32]. However, the inherent variability of the material obtained from different donors and the amount of material required [21] means that performing drug screens using this model is not a practical option.

**Fig 1:**
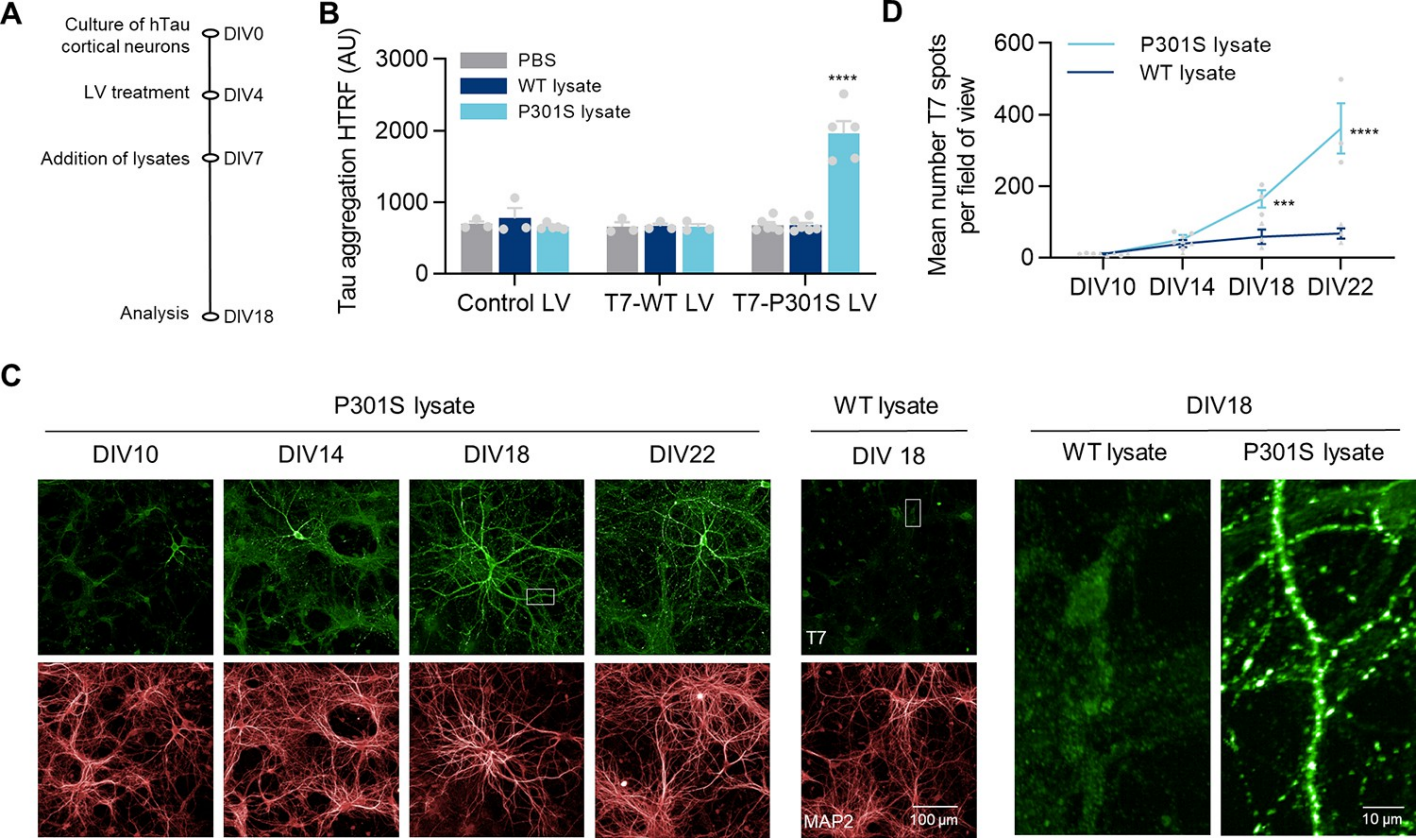
Designing a neuronal seeding-based model of tau accumulation. **A)** Diagram of experimental design. **B)** Tau aggregation HTRF in DIV18 lysates from cells treated with control, T7-WT tau or T7-P301S tau LV at MOI 5, and lysates from WT or P301S mice at 166pg/cell, or PBS as a vehicle control. Data shown as mean ± SEM, n = 3–4 independent experiments. Assay background = 627 ± 50. One-Way ANOVA with Dunnett’s multiple comparisons test. All conditions tested against Control LV + PBS. **C)** Representative images from hTau primary neurons treated with T7-P301S LV and WT or P301S brain lysates. Cells were fixed at the indicated DIVs. Left: zoom images of the areas highlighted in white. **D)** Quantification of the number of Triton-insoluble T7 inclusions in hTau primary neurons treated as in C. Data shown as mean ± SEM, n = 3–4 independent experiments. Two-Way ANOVA with Sidak’s multiple comparisons test, comparing WT vs P301S brain lysate for each DIV.

We focused on establishing a seeding-based neuronal model by incubating primary cortical hTau neurons with lysates from the cortex of 9 month-old P301S mice (Fig 1), as these lysates have been previously shown to facilitate the formation of tau inclusions in P301S animals *in vivo* and in HEK cell models of tau pathology [15, 24]. The highest amount of mouse cortical lysates added to hTau primary neurons from DIV7, in a single treatment and without causing toxicity during an 11-day treatment, was 3 μg of total protein (S3 Fig). This is equivalent to what we defined as a multiplicity of seeding (MOS) of 166 pg of protein per cell (166 pg/cell).

In these experimental conditions, we observed that P301S lysates were not able to induce the accumulation of humanized WT tau endogenous to hTau primary neurons (data not shown). Thus, to facilitate tau accumulation and the identification of newly formed inclusions, neuronal cultures were incubated with lentivirus (LV) that introduce human 2N4R tau fused to a T7 tag, with or without a P301S mutation (T7-P301S and T7-WT tau, respectively). LV expression, at MOI5 from day 4 in culture, lead to homogenous expression of T7-tau (S4 Fig). Tau aggregation HTRF showed that both the expression of T7-P301S tau and the addition of seeds from P301S animals were required to induce tau accumulation in this model by DIV18 (Fig 1B). Note that overexpression of T7-WT tau, even in the presence of seeds from P301S animals, did not lead to the accumulation of tau. Specific immunofluorescence (IF) analysis confirmed the presence of Triton-insoluble tau inclusions at DIV18 in hTau neurons. Cells with insoluble tau were identified by their high T7 labelling, and small puncta with high levels of insoluble T7-immunoreactivity were found along axons (Fig 1C, S5 Fig). Kinetic experiments indicated that the number of T7-P301S tau inclusions increased in a time-dependent manner, starting at DIV14, 7 days after treatment with P301S lysates (Fig 1C and 1D).

### Markers of tau pathology

**Fig 2:**
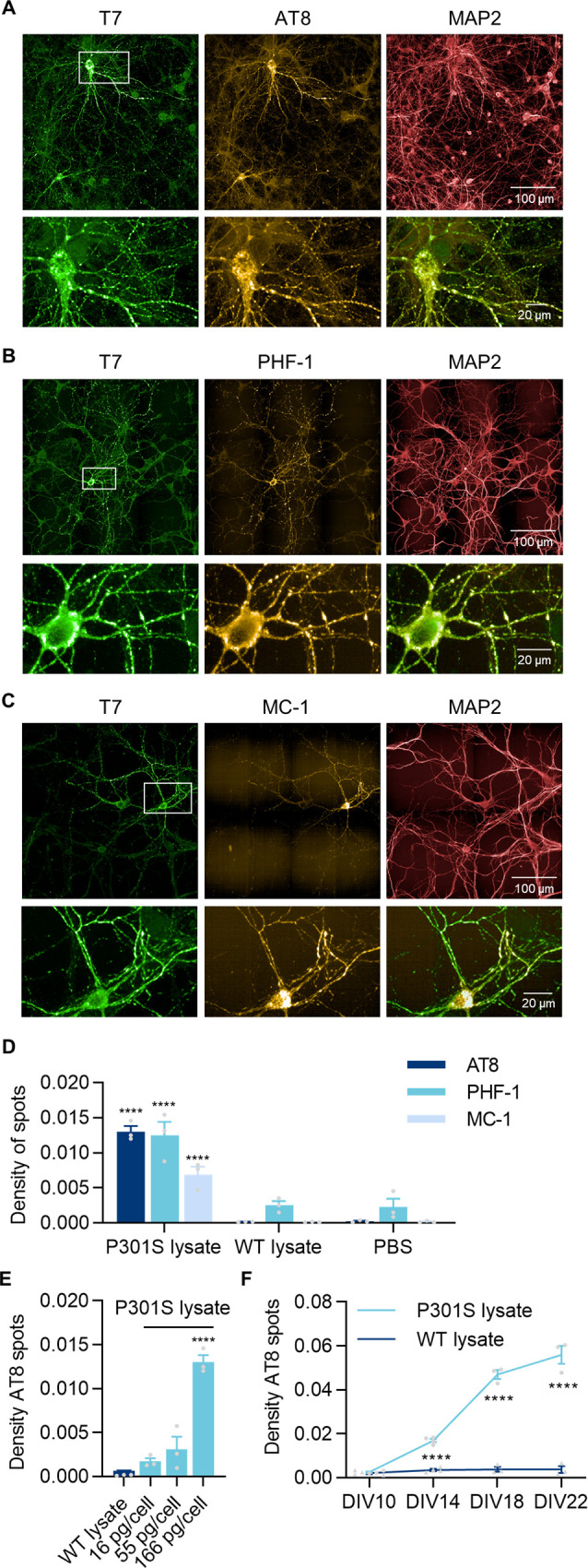
Triton-insoluble tau is positive for markers of tau pathology. Representative images from hTau neurons transduced with T7-P301S LV at DIV4, treated with P301S lysates at DIV7 and fixed at DIV18. Immunofluorescence for T7 (green), MAP2 (red) and markers of tau pathology (yellow) AT8 (**A**), PHF-1 (**B**) and MC-1 (**C**). **D)** Quantification of the density of Triton-insoluble AT8/PHF-1/MC-1 spots in hTau neurons transduced with T7-P301S LV and treated with PBS, WT or P301S lysates. Data shown as mean ± SEM, n = 3 independent experiments. Two-Way ANOVA with Sidak’s multiple comparisons test. For each marker, all conditions were tested against PBS control. **E)** Quantification of the density of Triton-insoluble AT8 spots in cells transduced with T7-P301S LV and treated with WT lysate at 166 pg/cell, or P301S lysate at the indicated concentrations. Data shown as mean ± SEM, n = 3 independent experiments. One-Way ANOVA with Dunnett’s multiple comparisons test. All conditions compared with WT lysate. **F)** Quantification of the density of Triton-insoluble AT8 spots in cells transduced with T7-P301S LV and treated with WT or P301S lysate at 166 pg/cell. Data shown as mean ± SEM, n = 3–4 independent experiments. Two-Way ANOVA with Sidak’s multiple comparisons test, comparing WT vs P301S lysate for each DIV.

To further characterize the insoluble T7-P301S tau inclusions we performed a series of IF experiments with antibodies against widely used markers of pathological tau species [33]. Triton-insoluble T7-P301S tau colocalised with puncta strongly positive for AT8, PHF-1 and MC-1 (Fig 2A–2C), and, at a weaker level, with AT180 (S6 Fig). Altogether, the presence of these markers is indicative of tau hyperphosphorylation and misfolding in the Triton-insoluble T7-P301S inclusions of our neuronal model.

In order to evaluate the extent of Triton-insoluble T7-P301S tau inclusions in culture, we developed a high content imaging analysis algorithm to quantify the density of spots (number of puncta per μm^2^ of neuronal area, see Methods), which are both T7-positive and tau AT8-, PHF-1- or MC-1-positive. We believe this is a better read-out than the number of T7 spots alone because it implies not only that there is formation of deposits of Triton-insoluble endogenous tau, but also that they are positive for known markers of tau pathology. In addition, the algorithm takes into account the total area of neurons in each condition to reduce variability due to the natural variation in cell numbers between culture wells, or due to variations in cell growth when comparing results from different time-points.

Analysis of the density of spots with all three markers of tau aggregation was able to clearly differentiate “seeded” from “non-seeded” conditions (Fig 2D). Analysis with MC-1, however, showed a reduced density of spots than for AT8 and PHF-1 antibodies. This was due to the lower intensity of this marker in puncta along neurites, which made their automated identification more difficult. Though the density of AT8 and PHF-1 spots was identical, because the PHF-1 antibody had a higher background, we determined the AT8 immunoreactivity to be the best read-out for further studies. Treatment with P301S seeds induced a MOS-dependent increase in the density of Triton-insoluble AT8 spots, with a maximum at 166 pg/cell (Fig 2E). In addition, kinetic experiments confirmed the time-course of the formation of phospho-tau spots (Fig 2F), as previously observed with T7 staining alone.

Altogether, we established the conditions of the model to be used in further studies: unless otherwise stated, hTau primary neurons were treated with T7-P301S LV (MOI 5) at DIV4 and with 166 pg/cell of P301S lysate at DIV7, up to DIV18. WT lysates were used as a negative control.

### Influence of different brain lysates in the levels of tau accumulation

When considering the reproducibility and consistency of our cellular model, one of the major sources of inter-experimental variability is the source of tau seeds. Preliminary experiments have shown that lysates from distinct animals added at the same MOS, though all capable of inducing tau accumulation, resulted in read-outs of varying amplitudes. Thus, we performed a series of experiments to establish if the source of the observed variability arose from the overall capacity of the lysates of promoting endogenous tau accumulation, and/or from the rate at which they do so.

**Fig 3:**
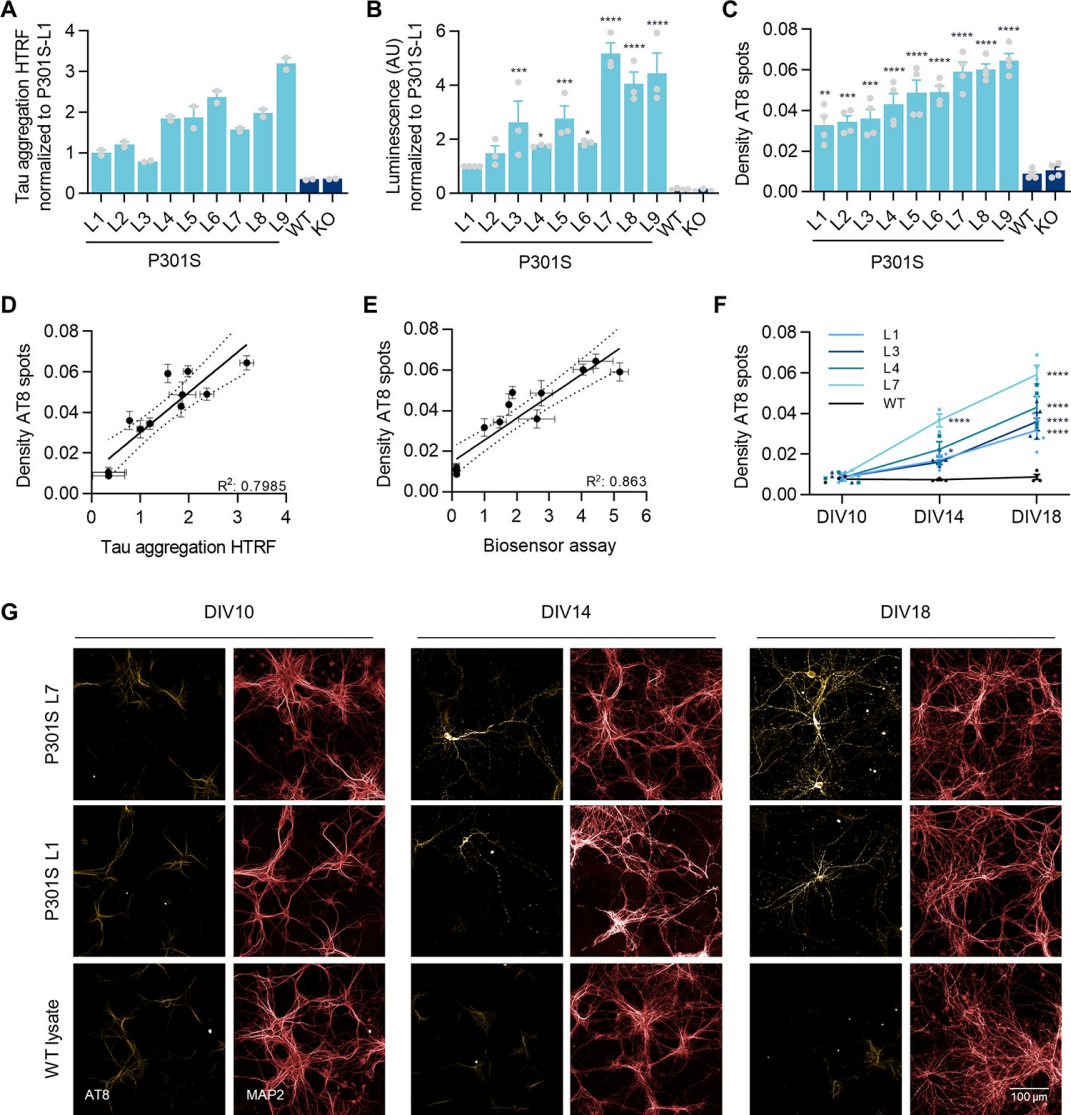
P301S lysate seeding potency correlates with formation of tau inclusions in seeded neurons. **A)** Levels of accumulated tau, as measured by Tau aggregation HTRF assay, in brain lysates from WT, KO and various P301S animals (9 P301S lysates identified as L1-L9). For ease of comparison, raw values were normalised to the value of the P301S lysate with the lowest signal (P301S L1). Data shown as mean ± SEM, n = 2 technical replicates. **B)** Seeding potency measured by luminescence-based Tau Biosensor Assay using the same lysates as in (A). Raw values were normalised to the value of the P301S lysate L1. Data shown as mean ± SEM, n = 3–4 independent experiments. One-Way ANOVA with Dunnett’s multiple comparisons test. All conditions compared with WT lysate. **C)** Density of Triton-insoluble AT8 spots in hTau primary neurons transduced with T7-P301S LV and treated with the lysates form (A). Data shown as mean ± SEM, n = 3–4 independent experiments. One-Way ANOVA with Dunnett’s multiple comparisons test. All conditions compared with WT lysate. **D-E)** Linear regressions between the parameters Density of AT8 spots and Tau aggregation HTRF (D) or results of the Biosensor Assay (E). Dotted lines represent 95% confidence interval. **F-G)** Density of Triton-insoluble AT8 spots and representative images from hTau primary neurons transduced with T7-P301S LV and treated with a selection of lysates from (A). Cells were fixed at the time-points indicated in the graph. Data shown as mean ± SEM, n = 3 independent experiments. Two-Way ANOVA with Sidak’s multiple comparisons test, comparing WT vs P301S lysates for each DIV.

Firstly, the comparison of aggregated tau levels of various P301S lysates prepared from nine individual mice (L1 to L9) showed a 3-fold variation in signal detected by Tau aggregation HTRF, indicating the presence of varying levels of P301S tau inclusions in the samples (Fig 3A). Next, the seeding potency of these P301S lysates was tested in a HEK293 cell tau Biosensor Assay. This assay also showed a range of responses between different lysates, which was indicative of varying seeding potencies for each sample (Fig 3B). Finally, in the primary neuron model, each lysate at the same MOS induced different densities of AT8 spots at DIV18 (Fig 3C), while the mean size of the inclusions was identical and neuronal health was unaffected (S7 Fig). In both assays, we did not detect any signal with the two negative controls, WT and KO brain lysates. Further analysis indicated that Tau aggregation HTRF and the Biosensor Assay correlated well with the density of Triton-insoluble AT8 spots at DIV18 (Fig 3D and 3E), indicating that both techniques can be used to predict the relative density of AT8 inclusions in hTau primary neurons and pre-select the most suitable brain lysates to be used in later applications.

To investigate the effect of seeding potency on the tau accumulation kinetics in hTau neurons, we tested a selection of four P301S lysates with different seeding potencies (L1, L3, L4 and L7). Despite different densities of AT8 spots at DIV18, the formation of tau puncta followed the same trend–spots were first seen at around DIV14 and double in quantity by DIV18 (Fig 3F and 3G).

### Second generation seeding

**Fig 4:**
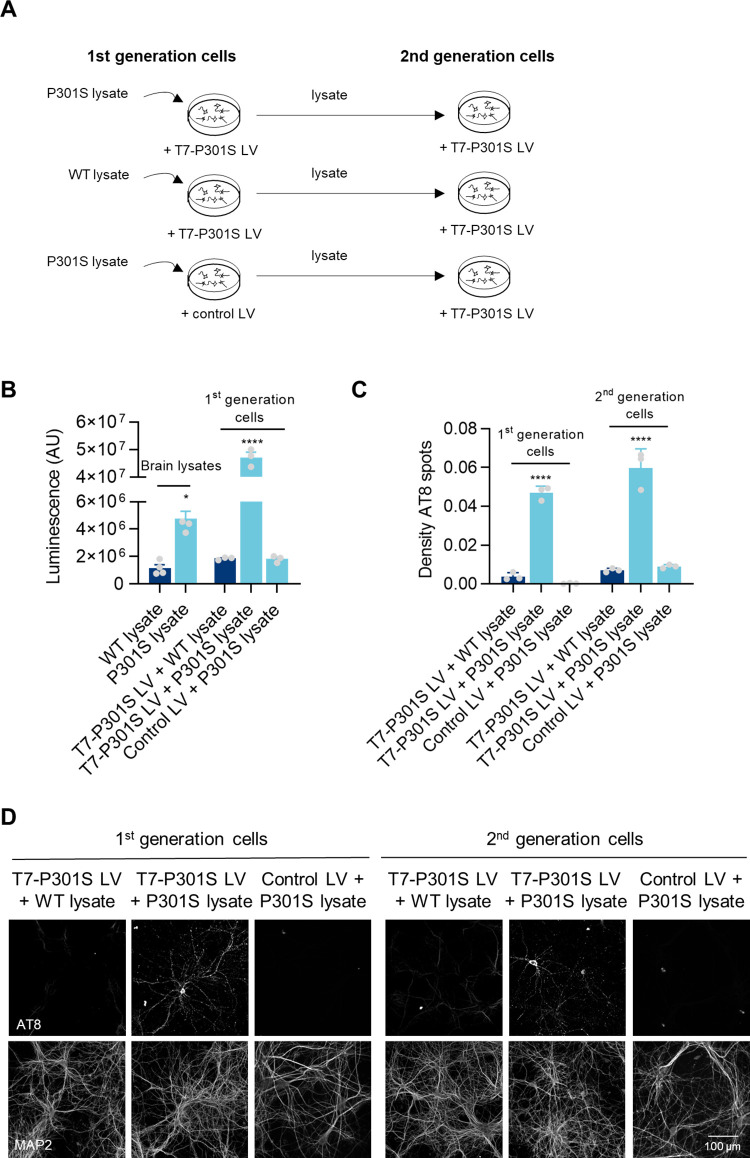
Second generation seeding. **A)** Diagram of experimental design. 1^st^ generation of hTau primary neurons were transduced at DIV4 with T7-P301S LV or Control LV at MOI 5 and incubated at DIV7 with 166.6 pg/cell of WT or P301S lysates, up to DIV18. 2^nd^ generation of hTau primary neurons were transduced at DIV4 with T7-P301S LV at MOI 5 and incubated at DIV7 with lysates of 1^st^ generation seeded hTau primary neurons at MOS 166.6 pg/cell, up to DIV18. **B)** Biosensor Assay measuring original WT and P301S lysates (brain lysates) and lysates from the 1^st^ generation of hTau primary neurons (1^st^ generation cells). Data shown as mean ± SEM. Brain lysates: n = 4 technical replicates. 1^st^ generation cell lysates: n = 3 independent cultures. One-Way ANOVA with Dunnett’s multiple comparisons test. All conditions compared with WT lysate. **C-D)** Density of AT8 spots and representative images of 1^st^ and 2^nd^ generation seeded hTau primary neurons (1^st^ generation cells and 2^nd^ generation cells, respectively). Data shown as mean ± SEM, n = 3 independent cultures. One-Way ANOVA with Dunnett’s multiple comparisons test. All conditions compared with T7-P301S LV + WT lysate, within each generation of cells.

The concept of seeding requires not only that a misfolded protein has the capacity to interact with native proteins and act as a template, converting them to its abnormal conformation, but also that the resulting aberrant proteins must also have the capacity to act as new seeds. To test if this was the case in our model, we collected and lysed 1^st^ generation of P301S-seeded primary neurons at DIV18 and evaluated their seeding potency and their capacity to induce tau inclusions in a 2^nd^ generation of hTau neuronal cultures overexpressing T7-P301S (Fig 4). Biosensor Assay indicated that the lysates of the 1^st^ generation of P301S-seeded cells (T7-P301S LV + P301S lysate) exhibited seeding activity, while lysates from cells incubated with WT lysates (T7-P301S LV + WT lysate) did not. Similarly, only the P301S-seeded cells, when incubated in a new neuronal culture expressing T7-P301S, recreated the AT8 inclusion phenotype. Importantly, we performed an additional control experiment (Control LV + P301S lysate) to verify that the detected seeding capacity of the lysates of the 1^st^ generation of P301S-seeded cells was not due to residual activity from traces of the original P301S brain lysate. No positive signal was found in the two assays tested, indicating that new seeds were being propagated. Altogether, these data demonstrated that our seeding neuronal model is able to create seeding-competent species.

### Strategies to prevent the formation of tau inclusions

Having established the conditions necessary to induce the formation of tau inclusions in hTau primary neurons, we tested how the model responded to molecules aimed at preventing tau aggregation, as these are the focus of many therapeutic approaches for Tauopathies.

**Fig 5:**
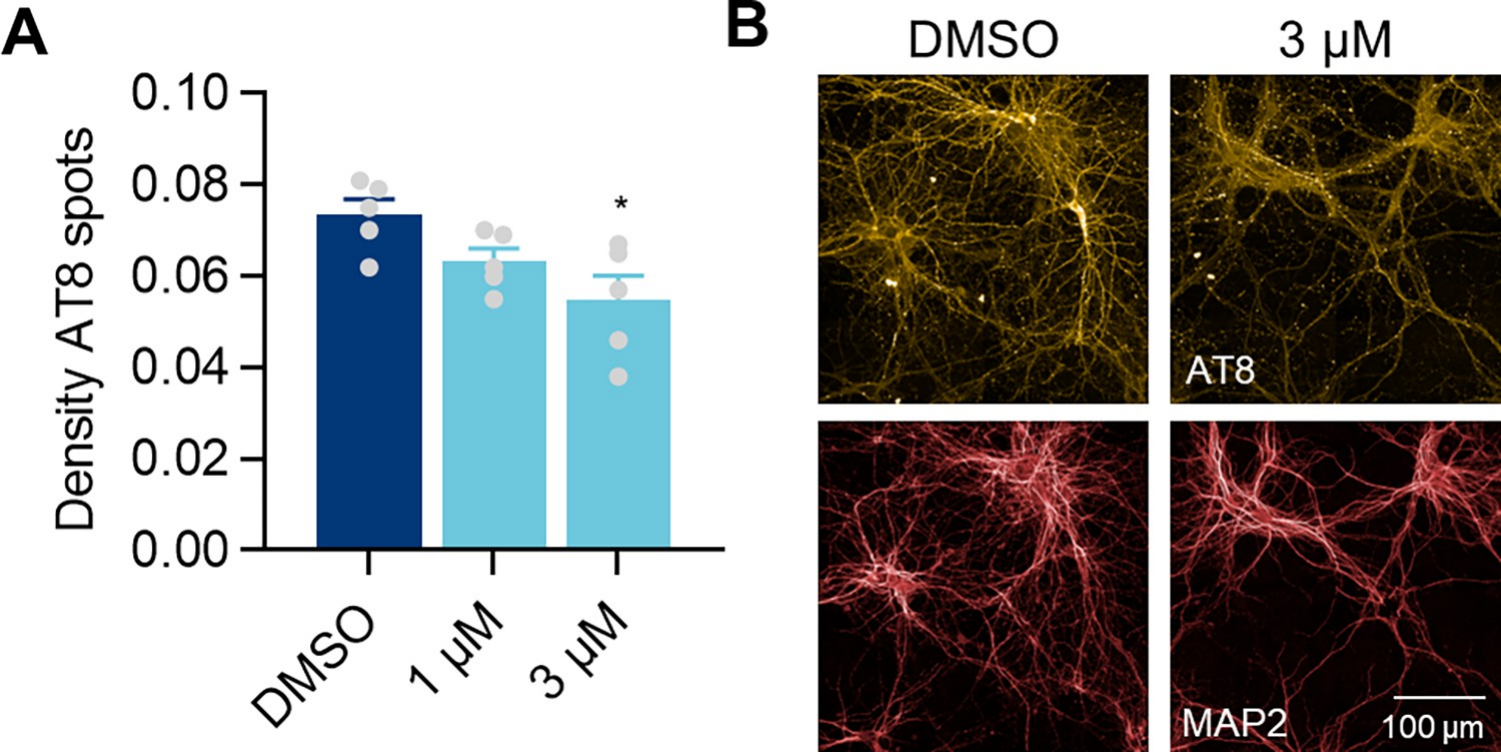
Anle138b prevents tau accumulation in seeded primary neurons. **A)** Density of Triton-insoluble AT8 spots and representative images (**B**) from hTau primary neurons transduced with T7-P301S LV at DIV4 and treated with P301S lysates at DIV7, in the presence of 1 μM and 3 μM of the anti-aggregation compound Anle138b from DIV6. DMSO was used as vehicle. Data shown as mean ± SEM, n = 5 independent experiments. One-Way ANOVA with Dunnett’s multiple comparisons test. All conditions compared with DMSO control.

The oligomer modulator Anle138b has been shown to reduce tau aggregation and delay disease progression in mouse models of Tauopathies [34, 35]. Therefore, we tested if this compound could also reduce the accumulation of T7-P301S tau in our cellular model. Treatment with 3 μM of Anle138b, from DIV6 over the course of 12 days, before the formation of T7-P301S inclusions, resulted in a small but significant decrease in the density of Triton-insoluble AT8 spots in seeded primary neurons (Fig 5).

**Fig 6:**
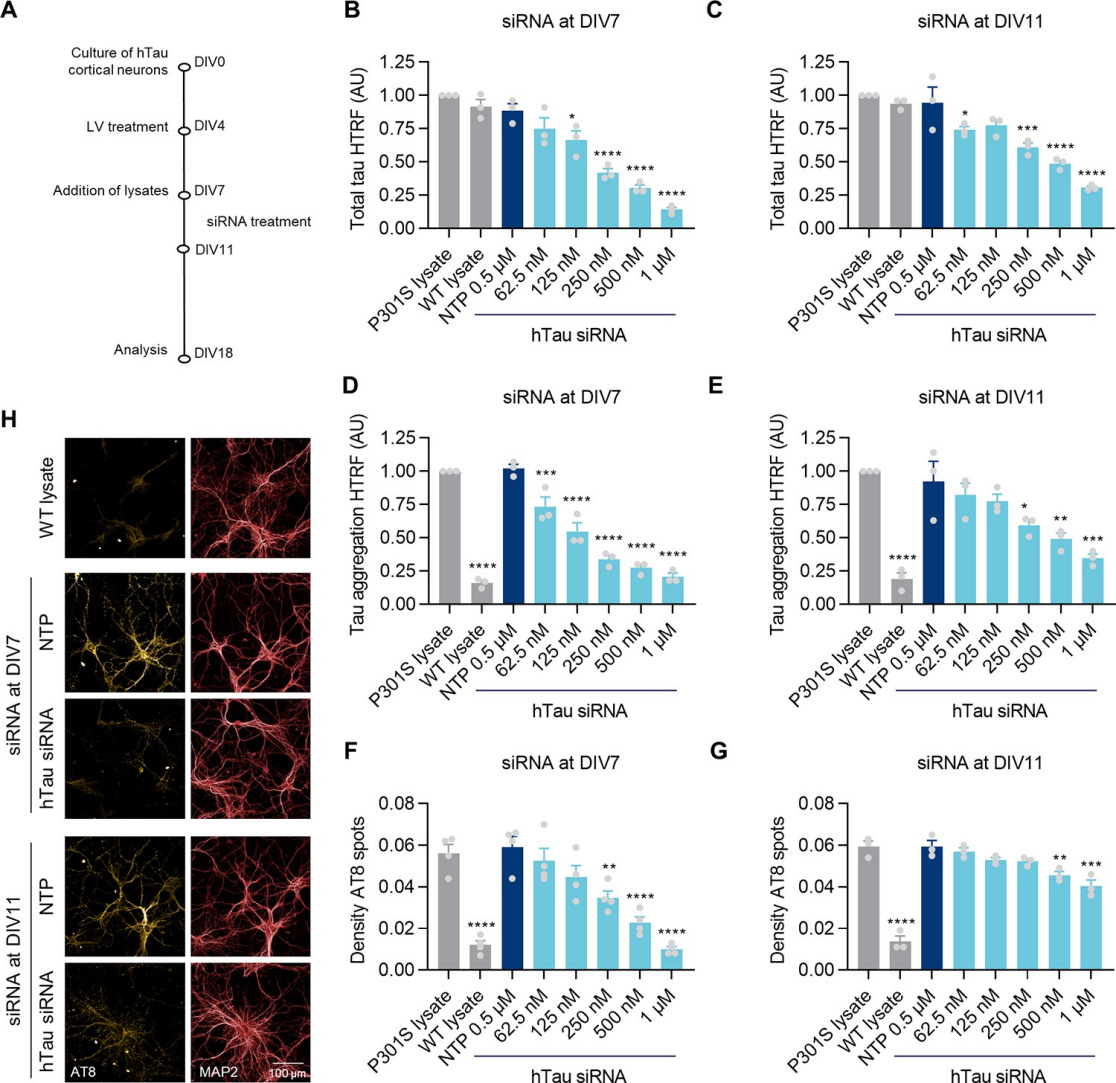
Human tau knock-down by siRNA decreases tau inclusions in seeded hTau primary neurons. **A)** Diagram of experimental design. hTau primary neurons transduced with T7-P301S LV at DIV4 were treated with WT or P301S lysates at DIV7 and increasing concentrations of human hTau siRNA, from either DIV7 or DIV11. Non-targeting pool (NTP) siRNA at 0.5 μM was used as a control. Cells were lysed for HTRF assay or fixed for immunofluorescence at DIV18. Total tau HTRF of seeded primary neurons treated with siRNA at DIV7 (**B**) or DIV11 (**C**). Accumulated tau as measured by Tau aggregation HTRF (**D-E**) or Triton-insoluble AT8 spots as quantified by IF (**F-G**). Grey bars: treatment with T7-P301S and WT or P301S lysates, without siRNA. Blue bars: treatment with T7-P301S LV, P301S lysate and siRNA as indicated in the graph. Data shown as mean ± SEM, n = 3 independent experiments. One-Way ANOVA with Dunnett’s multiple comparisons test, all conditions compared with NTP treatment. **H**) Representative images from hTau neurons treated as in (F-G) with 0.5 μM of NTP or hTau siRNA.

Additionally, we tested human tau siRNA (hTau siRNA) as a proxy for therapies aimed at reducing tau mRNA levels (Fig 6). We chose two time-points for applying the siRNA treatment in increasing concentrations to the primary neuron seeding model: treatment from DIV7, to capture the effect of intervening in the early stages of tau inclusion formation; and treatment from DIV11, to evaluate the effect of reducing tau levels once the presence of inclusions is already established.

The hTau siRNA treatment at DIV7 and DIV11 reduced tau protein levels in a dose-dependent manner in seeded neurons, as confirmed by HTRF for total cellular tau (Fig 6B and 6C). In addition, we measured the level of tau accumulation by Tau aggregation HTRF and the density of Triton-insoluble AT8 spots in neuronal cultures by IF analysis (Fig 6D–6H). Early siRNA intervention abolished the formation of tau inclusions by reducing the levels of tau protein by ~75%, using 0.5–1 μM of hTau siRNA. Lower concentrations of hTau siRNA (125–250 nM), that reduced tau levels by only around 25–50%, also led to a strong reduction in the level of tau inclusions by DIV18. A shorter treatment with hTau siRNA from DIV11 resulted in a significant, though milder, effect which required 0.5–1 μM of hTau siRNA to reduce tau inclusions by ~25–50% within 6 days of treatment. Overall, these data show a strong and fast effect of decreased levels of tau protein in reducing tau accumulation in our seeded neuronal model.

## Discussion

Tau aggregation is believed to be at the centre of disease progression in AD, PSP and other Tauopathies [5, 6, 9, 36]. As a result, many efforts have been made to develop experimental models of tau aggregation to improve our understanding of the mechanisms leading to the initiation and progression of tau pathology, as well as to support the development of novel therapies. However, achieving consistent tau aggregation is not straightforward—tau is an intrinsically disordered protein not prone to aggregate spontaneously [37, 38]. Nonetheless, some experimental conditions can promote its aggregation, such as through the addition of seeds and/or the over-expression of tau fragments (eg. tau repeat domain) or of tau variants with disease-associated mutations (eg. P301S or P301L). In this study, we explored the use of seeds derived from brain lysates from aged transgenic P301S mice (also known as PS19 mice) [31]. These animals develop progressive tau pathology which, by 9 months of age, is well established across several brain regions. Lysates from those regions are able to seed tau aggregation in HEK293 cells and mouse models [15, 16, 18, 23].

We observed that achieving tau accumulation in mouse hTau primary neurons required the presence of the P301S mutation in both the intracellular and seed-competent tau. Lysates from P301S animals did not promote accumulation of (over)expressed WT hTau, indicating that endogenous tau must present a specific conformation, provided by the P301S mutation, to be transformed by P301S mouse-derived seeds. This is in agreement with previous studies, which showed similar requirements in matching tau variants between seed-competent and monomeric tau [19, 25, 39]. Interestingly, in the same hTau primary neurons, PHFs extracted from samples from PSP donors were able to induce the accumulation of WT hTau. These data suggest a higher molecular complexity of the human-derived samples, potentially with diverse variants and tau conformations contributing to its seeding capacity [40, 41].

Using IF, we detected the presence of well-defined intraneuronal inclusions of Triton-insoluble tau immunoreactive for AT8, PHF-1 and MC-1 antibodies. These markers recognize variants of phosphorylated (AT8: Ser202/Thr205; PHF-1: Ser396/Ser404) and misfolded (MC-1) tau that are characteristic of neurofibrillary tangles [33]. By quantitative analysis, we observed that AT8 immunoreactivity provided the more consistent data across experiments. In addition, it is important to emphasise the need for removing soluble tau during the fixation process [17, 20]. This technique allows the differentiation of tau inclusions from the ubiquitous intracellular signal of phosphorylated and, to a lesser extent, MC-1 positive tau. Indeed, phosphorylated forms of the protein, as is the case for AT8, PHF-1 and AT180, are highly present in hTau neurons where tau is overexpressed (S6 Fig). Although some conformation-dependent markers, such as MC-1, can identify seeded cells using standard protocols, removal of the soluble component of tau results in more easily measured read-outs for most other markers.

Assessment of the kinetics of tau accumulation in our model revealed that a significant rise in the density of triton-insoluble AT8 spots was first detected at DIV14 in culture, 7 days after a single treatment with P301S lysates, and that the increase in the number of tau inclusions continued over time. The kinetics of appearance of tau accumulation was similar when lysates from distinct animals were used. Such consistency in the progression of tau pathology is key to the success of this model–it makes it possible to pool lysates from numerous animals without affecting the kinetics of tau accumulation, thus allowing large set of experiments to be performed using the exact same source of seeds throughout.

Another essential feature of this model is the capacity of the hTau neurons containing accumulated tau to act as seeding agents themselves. Importantly, only lysates from cells treated with P301S lysates and also originally expressing LV-P301S were able to transfer their seeding capacity. In addition, we have excluded the possibility that overexpression of T7-P301S alone or leftover seeds from the original P301S lysates were responsible for inducing Triton-insoluble AT8 positive tau inclusions in the second generation of seeded cells. This is the first time that the ability to serially propagate seeding-competent activity has been shown in hTau primary neurons using mouse brain derived seeds. Overall, the ability to stably propagate tau pathology across various generations of cells confirms the seeding nature of the P301S model. It would be interesting to see if the observed inclusions are structured tau aggregates.

Many therapies being developed for the treatment of tauopathies focus on managing tau pathology by i) targeting tau post-translational modifications, such as hyperphosphorylation; ii) preventing tau aggregation; iii) promoting clearance of existing aggregates by boosting cell’s protein degradation mechanisms or by directly targeting tau for degradation; and iv) reducing the pool of available tau. In practice, this can be achieved by a variety of strategies such as, but not limited to, the use of kinase inhibitors, anti-aggregation compounds, tau anti-sense therapies, and passive or active tau immunotherapies [5–7, 42]. Thus, we tested the suitability of the tau neuronal seeding model for use in pharmacology studies using two strategies: by reducing tau accumulation and by reducing tau expression.

Anle138b is an anti-aggregation compound that has been shown to reduce tau aggregation and disease progression in cellular and mouse models of tau pathology [34, 35, 43, 44]. We showed that prolonged treatment with Anle138b led to a decrease in the density of Triton-insoluble AT8 spots in our model, consistent with a reduction in the formation of new aggregates.

Many tau-targeted therapies focus on reducing the levels of tau protein. Therefore, we used human tau siRNA as a proxy to reduce the intracellular pool of tau. We showed that the exposure of hTau neurons to increasing concentrations of tau siRNA led to a dose-dependent reduction in the level of accumulated tau, as measured by both Tau aggregation HTRF and IF. The effect was particularly robust when the levels of tau were decreased early during the process, in which a ~75% reduction of tau protein levels abolished the formation of inclusions. Importantly, smaller reductions in the levels of tau or siRNA treatment at later stages, when tau inclusions were already present in culture, also resulted in strong beneficial effects.

**Fig 7:**
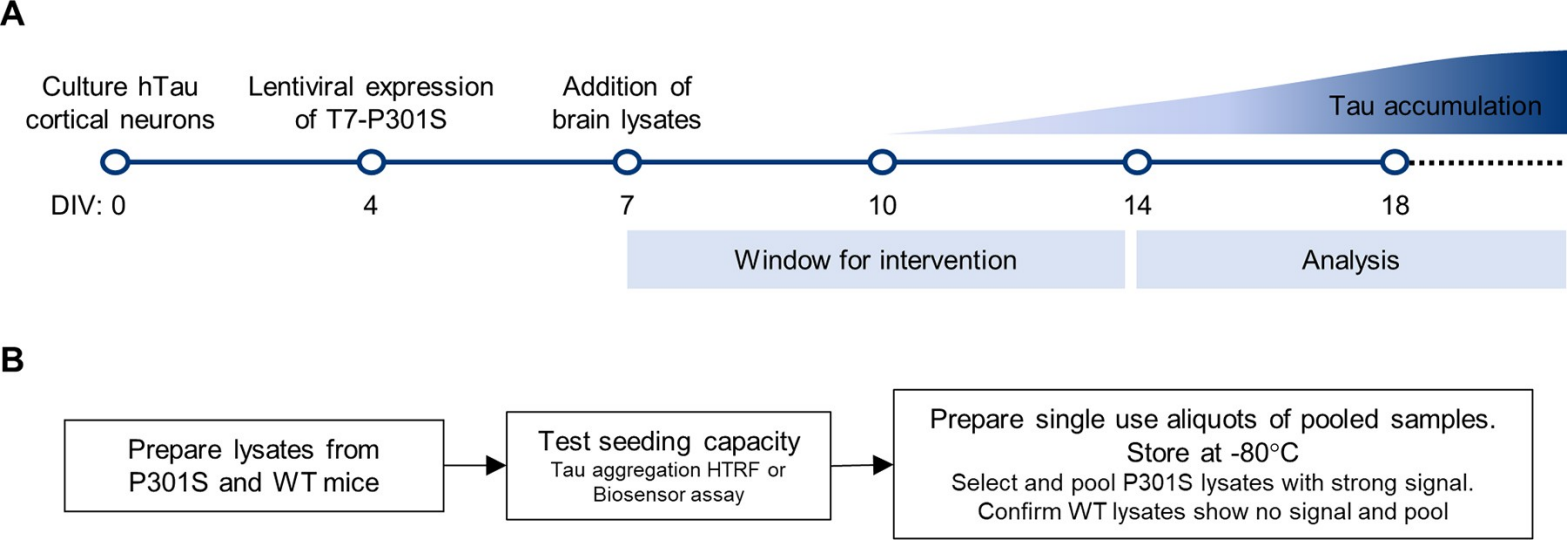
Implementation of the tau seeding model for drug screening. **A)** Diagram highlighting the key steps in the culture and treatment of primary neurons for use with the tau seeding model. Cortical neurons are cultured from hTau embryos, transduced with T7-P301S LV at DIV4 and treated with P301S lysates at DIV7. WT lysates are used as a negative control. Tau inclusions are detectable from DIV14 onwards. Treatments aimed at reducing the level of accumulated tau can be started from DIV7. The specific time-points of intervention and analysis may be defined by the end user according to experimental needs. **B)** Strategy for obtaining large quantities of comparable seeding material to reduce seeding variability.

Altogether, this tau seeding model can be used to simultaneously test the effects of new compounds on limiting tau seeding and accumulation, and on reducing tau levels, saving time and resources. In addition, the data suggests it is possible to slow down tau accumulation once tau pathology is already established, as would be the case in most clinical scenarios, thus broadening the therapeutic window available for testing of new drug candidates (Fig 7A).

Since we demonstrated that the differences in seeding potency of the lysates were correlated with the level of accumulated tau in our model, care must be taken when designing larger sets of experiments which require multiple rounds of independent, but comparable, individual experiments. To reduce the variability introduced by using different brain lysates, we recommend pooling lysates with efficient seeding activity when performing a batch of experiments that require a lot of seeding material. Pre-screening the lysates, by using Biosensor cells or Tau aggregation HTRF assays, will allow the most potent lysates to be combined (Fig 7B).

Regarding the cell type used, throughout this study we used hTau primary neurons for preparing mouse cortical cultures in order to maintain a consistent human tau background when using human PSP-derived seeds. Though we cannot exclude that mouse tau will interfere with P301S seed-induced inclusions [19], using WT neurons for the preparation of neuronal cultures may be a valuable option for many users.

When modelling tau aggregation, particularly for use in drug discovery, it is essential to try to mirror human pathology as closely as possible. One of the main ways of achieving this is by using patient-derived material as a source of tau seeds [15, 18–21, 45, 46]. However, the nature and isoform content of tau aggregates varies between tauopathies and is associated with clinical heterogeneity [36, 47–51]. Here, we prepared PHFs from the Globus Pallidus of PSP donors, as this region is one of the earliest to be affected in the course of PSP [52, 53], and used them to induce the formation of tau-tau interactions in the Biosensor assay, as well as to promote the formation of AT8 and MC-1 positive Triton-insoluble tau inclusions in hTau mouse primary neurons. We observed a high variability in the seeding potency of the material obtained from different donors, as generally described for human samples [19, 21, 54–56]. Interestingly, contrary to mouse P301S lysates, the seeding potency of human PSP seeds did not always correlate with tau inclusions in hTau neurons. Altogether, considering the amount of material required and the importance and scarcity of human tissue, we believe that performing large drug screens using human-derived seeds is not a practical, economical or ethical option. Studies using patient material are still undeniably important for understanding the pathophysiology of tauopathies. However, for drug discovery, we support that such studies should be limited to confirmatory experiments of a small selection of candidates which have already been strongly validated by other methods, such as our seeding strategy described herein.

## Conclusions

In this study we developed a novel seeding-based neuronal model of tau accumulation, using humanized tau mouse cortical neurons and mouse-derived seeds. Tau inclusions were obtained by treating neurons overexpressing T7-P301S human tau with cortical lysates from aged P301S mice, and the accumulation of tau was detected and quantified by biochemical (HTRF) and imaging (IF) methods. This model is particularly useful for drug discovery because it uses a cellular system and tau seeds that are easy to prepare, are readily available in large quantities and are highly reproducible due to their homogenous source, thus allowing for a large number of compounds to be tested in comparable conditions. In addition, the specificities of the model can be adapted to accommodate the study of disease-specific tau mutations or the use of patient-derived material.

## Supporting information

S1 Raw images (PDF)

S1 File (DOCX)

S1 Fig HEK293 cell-based tau Biosensor assay. **A)** Scheme of the Nanoluciferase complementation-based biosensor of tau seeding. Biosensor HEK293 cells stably express K18(P301L) molecules fused either to the large (LgBit) or to the small (SmBit) moieties of the nanoluciferase enzyme, under the CMV or TK promoter, respectively. LgBit and SmBit do not have any affinity for each other. It is only in the presence of tau seeds that K18(P301L) molecules interact with each other (initial steps of aggregation), bringing together LgBit and Smbit and reconstituting the nanoluciferase enzyme. Upon addition of the enzyme substrate, the luminescence signal is emitted and correlates with K18 self-association. **B)** Biosensor signal in non-treated HEK293 cells (basal), and in cells treated with brain lysates from WT or P301S mice.
(TIF)

S2 Fig Preparation of Sarkosyl-insoluble fibrils from the Globus Pallidus of donors with Progressive Supranuclear Palsy. **A)** Table containing a summary of donor information. Control: non-affected donor. PSP: donor with Progressive Supranuclear Palsy. Last column indicates if the preparation of Sarkosyl-insoluble fibrils (or PHF—paired-helical filaments) was successful. PHFs prepared from donor Control 2 were very diluted and could not be used for further experiments. **B)** Tau aggregation HTRF in PHF preparations of control 1 and patients PSP1, 2 and 3. n = 1. **C)** Biosensor Assay performed in PHF preparations of control 1 and patients PSP1, 2 and 3. Mean ± SEM, n = 3 technical replicas. **D)** Quantification of Triton-insoluble AT8 spots in hTau neurons treated with PHFs at DIV7 and fixed at DIV20. Increasing concentrations of PHFs were used. Mean ± SEM, n = 3 technical replicas. **E)** Representative images of hTau primary neurons treated as in D. **F)** Zoom in in neurons treated with PHFs from donor PSP2.
(TIF)

S3 Fig Determining Multiplicity of Seeding (MOS). Phase images and Yoyo-3 (Red) of hTau primary neurons treated with increasing concentrations of lysates from WT or P301S animals. No LVs were used in these cultures. Cells were treated at DIV7 and followed until DIV18, in the presence of 300 nM of Yoyo-3 (Red, marker of dead cells). PBS was used as vehicle and 1 μM Staurosporine as a positive control to induce toxicity. Representative images from n = 4 independent cultures.
(TIF)

S4 Fig Expression of T7-WT or T7-P301S tau using lentivirus. **A)** Neurons transduced with lentivirus expressing T7-WT, T7-P301S or an empty virus (Control LV) at 5 MOI. LVs were added at DIV4 and cells fixed at DIV18. Examples images of cells fixed and labelled with antibodies against tau (green), T7 (yellow) and MAP2 (red) showing homogenous expression of T7-tau in the whole culture. **B)** Example of western blot images from lysates from cells treated at DIV6 for 7 days (see S1 Fig for uncropped western blots). **C)** Quantification of tau levels from B, normalised to beta-actin. Data shown as mean ± SEM, n = 3 independent cultures. One-Way ANOVA with Dunnett’s multiple comparisons test. All conditions tested against Control LV.
(TIF)

S5 Fig Example images of a large field of hTau neurons seeded with P301S lysate and immunolabelled for AT8 and T7. Cells were treated with T7-P301S LV at DIV4, 166 pg/cell of P301S lysates at DIV7, and fixed in the presence of 1% Triton-X 100 at DIV18. Note that AT8 colocalizes with T7 staining, though some T7 immunoreactivity does not show strong AT8 labelling (arrows).
(TIF)

S6 Fig Removal of soluble proteins by Triton-X 100. Example images of hTau neurons fixed with 4% PFA in the presence or absence of 1% Triton-X 100, to remove soluble proteins. All cells were treated with T7-P301S lentivirus at DIV4, 166 pg/cell of WT or P301S lysates at DIV7, and fixed at DIV18. IF was performed for T7 (green), MAP2 (red) and tau markers AT8/MC-1/PHF-1/AT180 (yellow). White asterisks provide location information for the zoomed areas on the left.
(TIF)

S7 Fig Characteristics of hTau primary neurons seeded with P301S or WT and KO control lysates. A) Neuronal area (μm^2^) of hTau neurons transduced at DIV4 with T7-P301S LV and treated at DIV7 with various P301S (9 P301S lysates identified as L1-L9), WT and KO brain lysates. Cells were fixed at DIV18 in the presence of 1% Triton. Data shown as mean ± SEM, n = 4 independent experiments, One-Way ANOVA not significant. B) Mean area of Triton-insoluble AT8 spots (pixel^2^) from neurons treated as in A. Lysates from different animals induced the formation of inclusions of the same size. Data shown as mean ± SEM, n = 4, One-Way ANOVA not significant. C) Example images of hTau neurons treated as in A.
(TIF)
