# Supplementary material for: A seeding-based neuronal model of tau aggregation for use in drug discovery
Source: PLoS One. 2023 Apr 4;18(4):e0283941. doi: 10.1371/journal.pone.0283941 (PMC10072482; doi:10.1371/journal.pone.0283941)

Original Gels for Supplemental Figure 2B

N=1

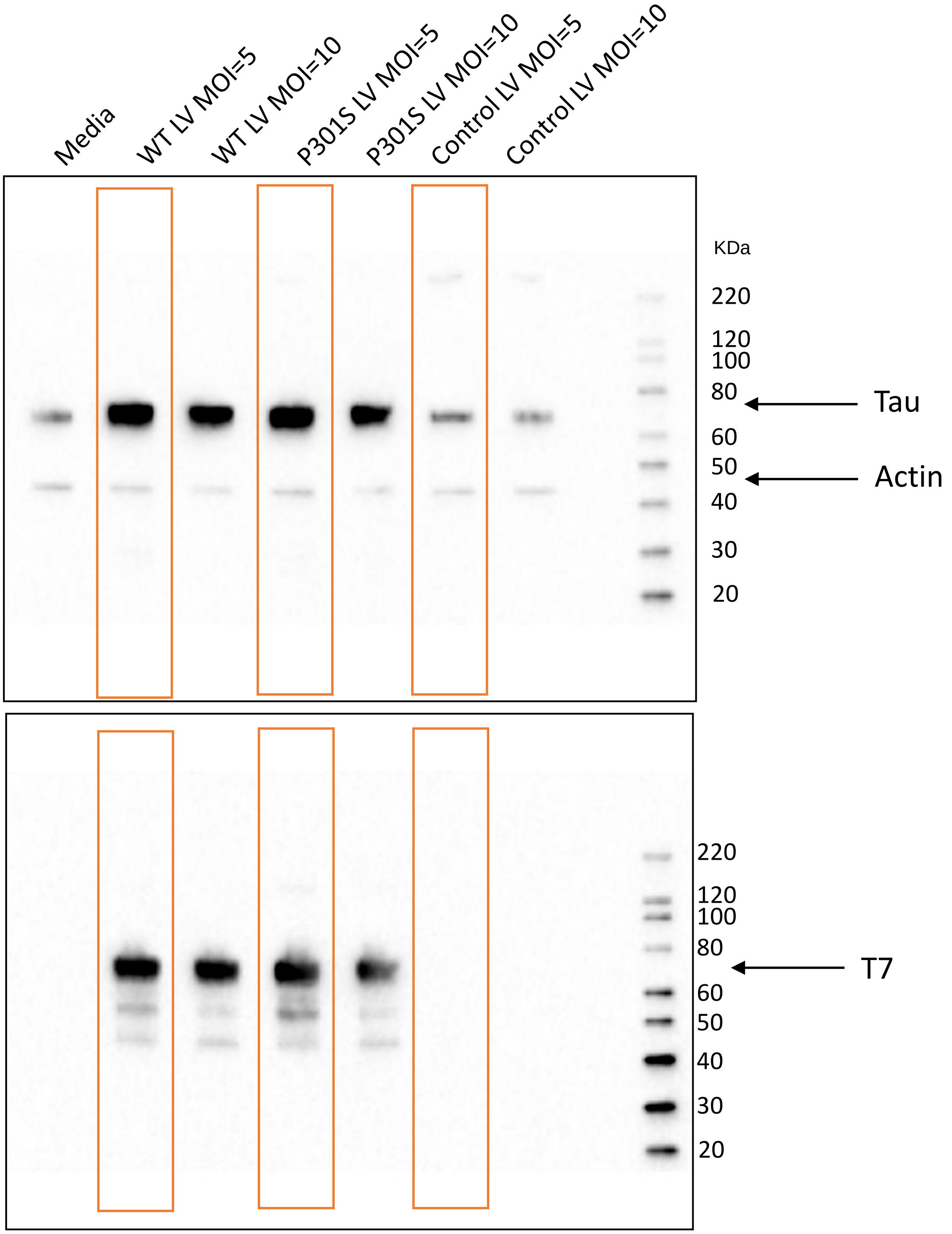

Original Gels for Supplemental Figure 2B

N=2

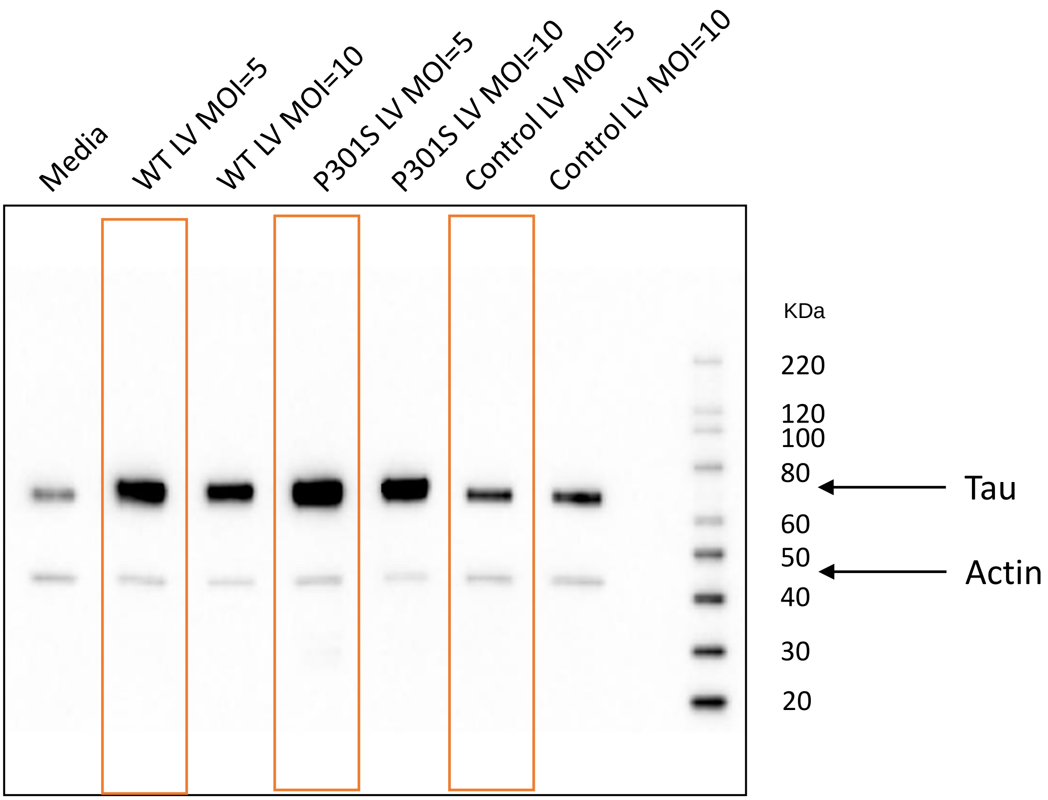

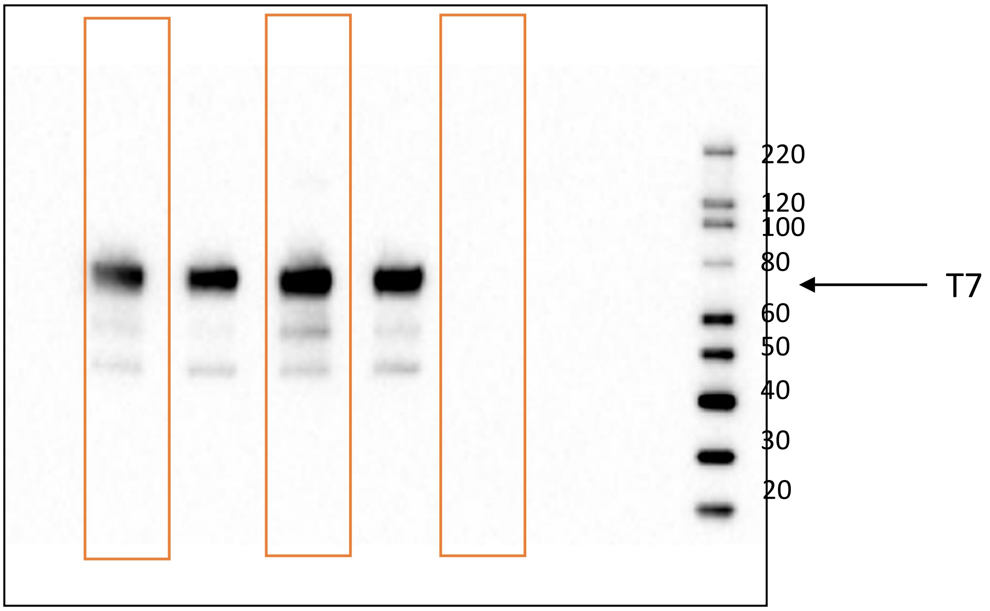

Original Gels for Supplemental Figure 2B

N=3

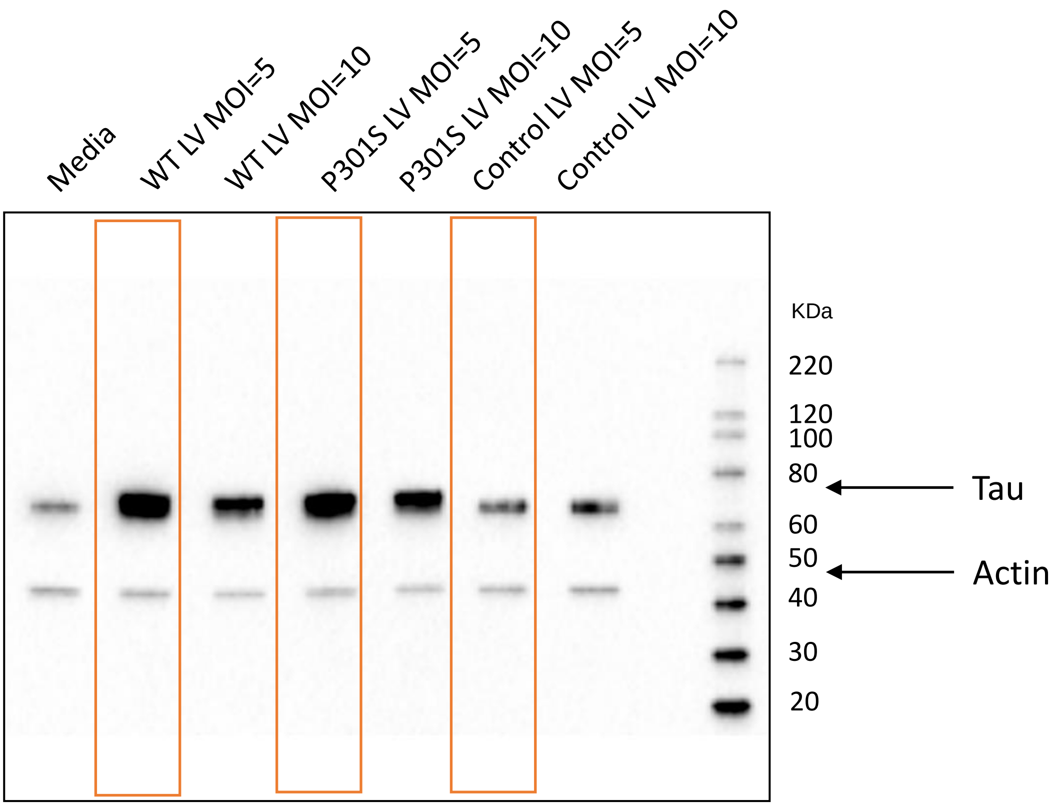

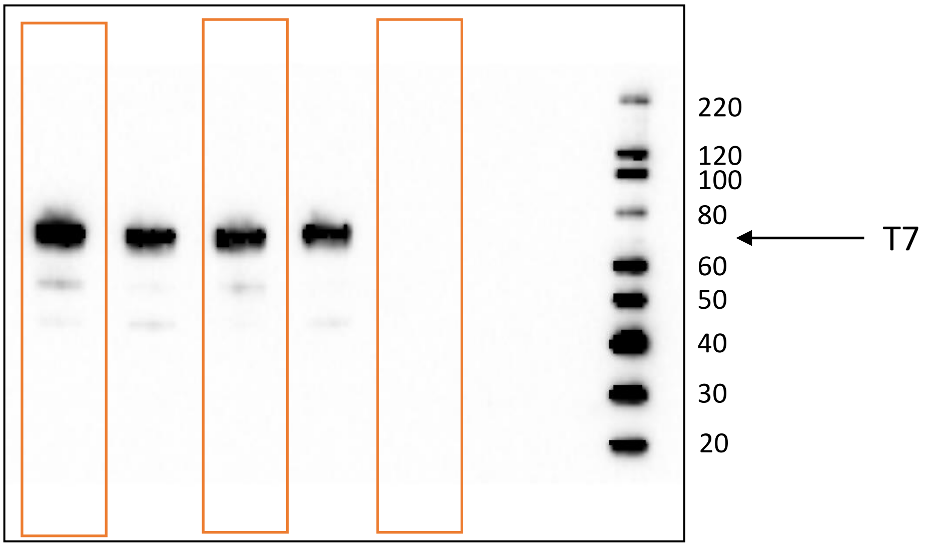

Supplement: S1 Raw images — (PDF) [file pone.0283941.s001.pdf]
