# Supplementary material for: A seeding-based neuronal model of tau aggregation for use in drug discovery
Source: PLoS One. 2023 Apr 4;18(4):e0283941. doi: 10.1371/journal.pone.0283941 (PMC10072482; doi:10.1371/journal.pone.0283941)
Supplement: S1 File — (DOCX) [file pone.0283941.s002.docx]

**Supporting Information – Additional Methods**

**Cellular toxicity**

Primary cortical neurons were treated at DIV7 with 300 nM YOYO^TM^-3 Iodide (Life Technologies), a cell-impermeant red fluorescent dye which is taken up only by dead cells. 1 µM Staurosporin (Sigma) was used as positive control of cell toxicity.

Cells were recorded live using the IncuCyte ZOOM™live-cell imaging system (Essen Bioscience) at 37°C and 5% CO2. The cell culture was imaged under phase-contrast and red fluorescence every 6 hours (4-9 images/well, 4 wells/condition), until up to DIV18.

**Western Blotting**

After 7 days of treatment with LVs, primary cells were lysed with with RIPA buffer, 1% proteases and phosphatase inhibitors and benzonase. Extracted proteins were reduced and denatured before being loaded at 8µg in commercially available gels were used for Bis-Tris 4–12% gradient gel (Life Technologies). After separation by electrophoresis, proteins were transferred to a nitrocellulose membrane. Blots were blocked in 5% dry milk blocking buffer (prepared in Tris-buffered saline with 1% Tween 20). Antibody dilutions were as follows: anti-T7 (1:10 000, GTX30555, Genetex); Tau 46 (1:2000, #4019, Cell Signaling); anti-actin 8H10D10 (1:1000, 12262, Cell Signaling); HRP anti-rabbit (1:2000, 7074 Cell Signaling),

and HRP anti-mouse (1:2000, 7076 Cell Signaling).

**Human samples**

Post-mortem frozen brain samples from patients with Progressive Supranuclear Palsy (PSP) and non-affected patients (Control) were obtained from Tissue Solutions (United Kingdom). Patient information was anonymized, and a brief clinical history was provided for each patient. Tissue was obtained from 2-3 patients per group, from the Globus Pallidus brain region. Sarkosyl-insoluble preparations, enriched in tau paired-helical filaments (PHFs), were prepared according to (Guo et al., 2016), sonicated (Q800R2, Sonica) at 4°C for 1 min at 90% power, divided in single use aliquots and stored at -80°C. Protein concentration was determined using a Pierce™ BCA Protein Assay Kit (ThermoFisher).

HTRF and Biosensor assay were performed as described in the main methods.

For seeding experiments, PHFs were added to mouse primary neurons (see main methods) at DIV7, at 16, 55 or 166 pg/cell. Cells were fixed at DIV20 with 4% PFA, 4% sucrose and 1% Triton-X 100 in PBS and processed for Immunofluorescence (primary antibodies: AT8 and MAP2) as described in the main methods section. For one experiment, neurons were additionally transduced with T7-WT LV at DIV4 and immunofluorescence was performed for T7, in addition to AT8 and MAP2.

Confocal imaging was performed using a 63x water objective in an Opera Phenix High-Content Screening System (PerkinElmer). z-stacks containing 5 1 µm z-planes, of 200 fields of view, were obtained for each well. Automated image analysis was set up for maximum intensity projections using the Harmony software (PerkinElmer). “Neuronal area” was defined as the area (µm²) of MAP2 in each well. The “density of AT8 spots” measurement was calculated, for each well, as the number of high intensity spots positive for AT8 normalised to the neuronal area (# spots/µm²).

**Biosensor assay**

The Human Tau K18 fragment containing the P301L mutation (amino acid sequence: QTAPVPMPDLKNVKSKIGSTENLKHQPGGGKVQIINKKLDLSNVQSKCGSKDNIKHV**L**GGGSVQIVYKPVDLSKVTSKCGSLGNIHHKPGGGQVEVKSEKLDFKDRVQSKIGSLDNITHVPGGGNKKIE) was inserted into the pcDNA6 (blasticidin resistant vector) downstream of the coding region of the LargeBit fragment, and pcDNA3.1+hygro (hygromicin resistant vector) downstream of the coding region of the SmallBit fragment of the nanoluciferase [32] by standard subcloning methods with restriction enzymes and T4DNA ligase [33]. LargeBit-K18(P301L)-pcDNA6(blast) and SmallBit-K18(P301L)-pcDNA3.1+(hygro) vectors were transfected in HEK293T cells simultaneously by Jet-PEI (Polyplus-transfection, New York, NY. After 48 hours, 50 µg/mL Hygromycin B and 5 µg/mL Blasticidin S were administrated into the medium. After three weeks of culture, double stable cell pools (referred to as Biosensor cells) were obtained.
