# Supplementary figures and images for: A seeding-based neuronal model of tau aggregation for use in drug discovery
Source: PLoS One. 2023 Apr 4;18(4):e0283941. doi: 10.1371/journal.pone.0283941 (PMC10072482; doi:10.1371/journal.pone.0283941)

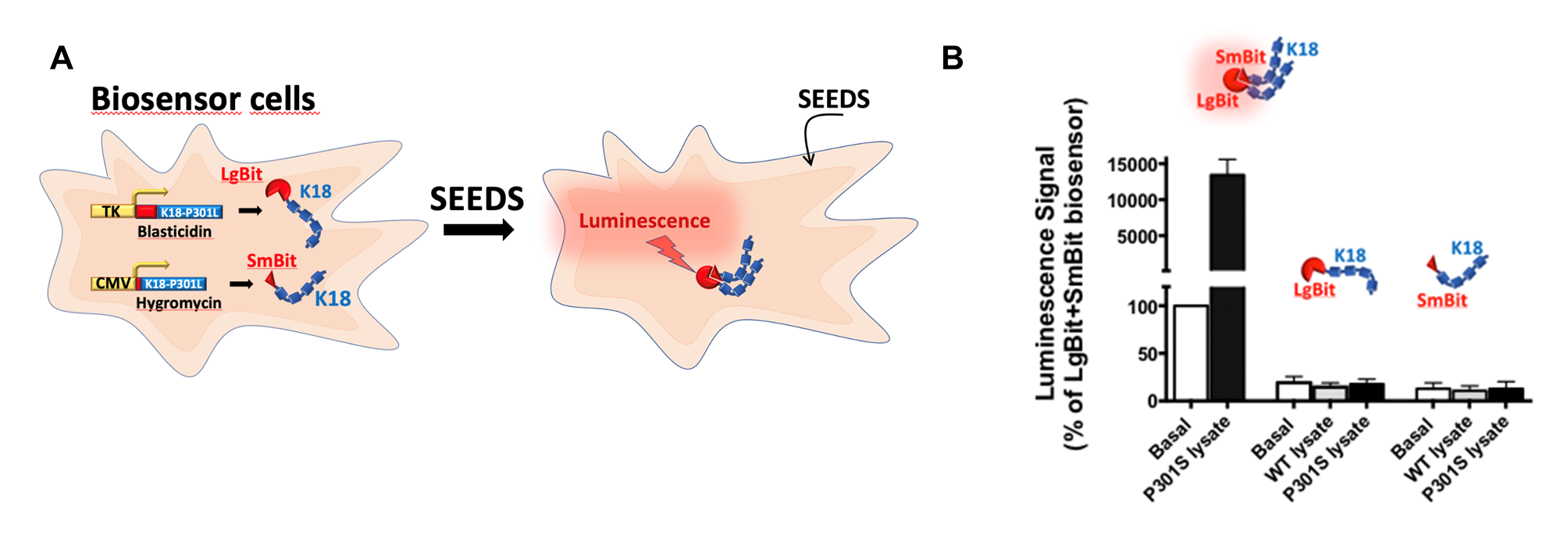

Supplement: S1 Fig — A) Scheme of the Nanoluciferase complementation-based biosensor of tau seeding. Biosensor HEK293 cells stably express K18(P301L) molecules fused either to the large (LgBit) or to the small (SmBit) moieties of the nanoluciferase enzyme, under the CMV or TK promoter, respectively. LgBit and SmBit do not have any affinity for each other. It is only in the presence of tau seeds that K18(P301L) molecules interact with each other (initial steps of aggregation), bringing together LgBit and Smbit and reconstituting the nanoluciferase enzyme. Upon addition of the enzyme substrate, the luminescence signal is emitted and correlates with K18 self-association. B) Biosensor signal in non-treated HEK293 cells (basal), and in cells treated with brain lysates from WT or P301S mice. (TIF) [file pone.0283941.s003.tif]

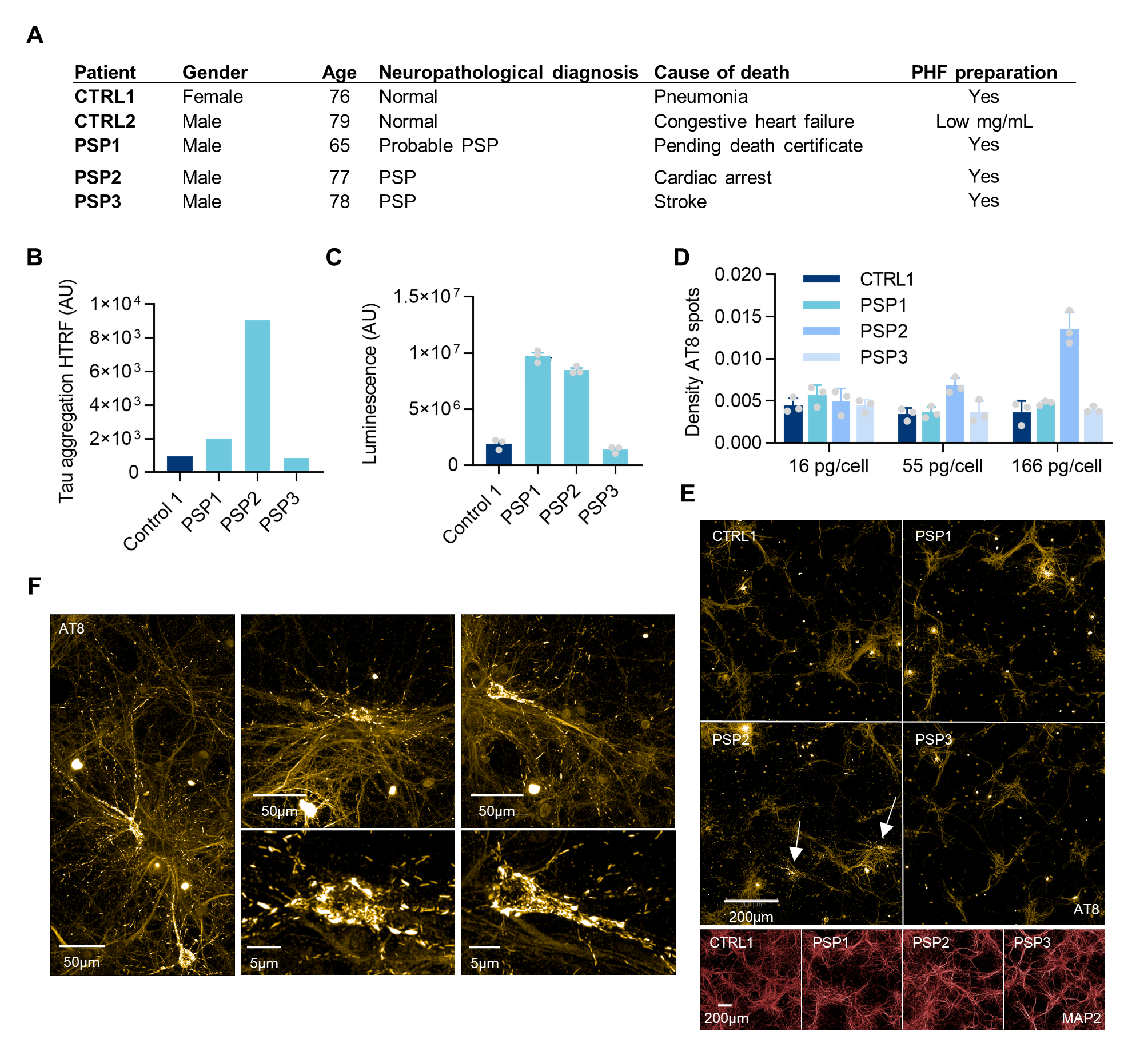

Supplement: S2 Fig — A) Table containing a summary of donor information. Control: non-affected donor. PSP: donor with Progressive Supranuclear Palsy. Last column indicates if the preparation of Sarkosyl-insoluble fibrils (or PHF—paired-helical filaments) was successful. PHFs prepared from donor Control 2 were very diluted and could not be used for further experiments. B) Tau aggregation HTRF in PHF preparations of control 1 and patients PSP1, 2 and 3. n = 1. C) Biosensor Assay performed in PHF preparations of control 1 and patients PSP1, 2 and 3. Mean ± SEM, n = 3 technical replicas. D) Quantification of Triton-insoluble AT8 spots in hTau neurons treated with PHFs at DIV7 and fixed at DIV20. Increasing concentrations of PHFs were used. Mean ± SEM, n = 3 technical replicas. E) Representative images of hTau primary neurons treated as in D. F) Zoom in in neurons treated with PHFs from donor PSP2. (TIF) [file pone.0283941.s004.tif]

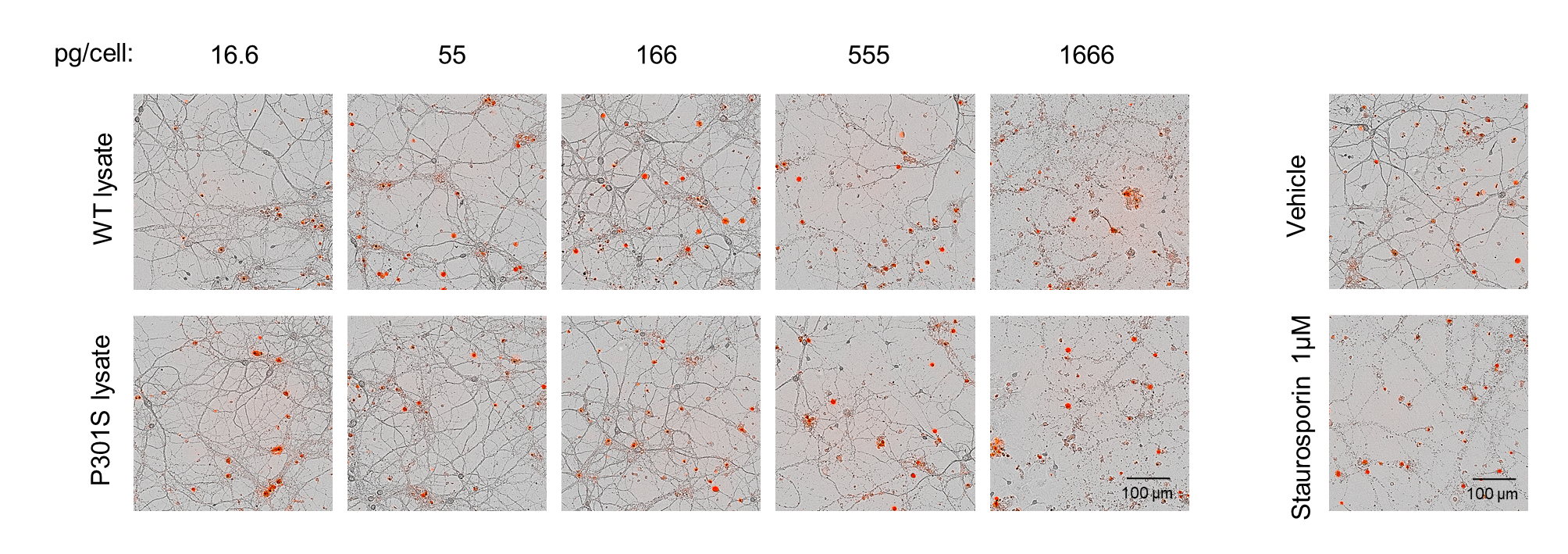

Supplement: S3 Fig — Phase images and Yoyo-3 (Red) of hTau primary neurons treated with increasing concentrations of lysates from WT or P301S animals. No LVs were used in these cultures. Cells were treated at DIV7 and followed until DIV18, in the presence of 300 nM of Yoyo-3 (Red, marker of dead cells). PBS was used as vehicle and 1 μM Staurosporine as a positive control to induce toxicity. Representative images from n = 4 independent cultures. (TIF) [file pone.0283941.s005.tif]

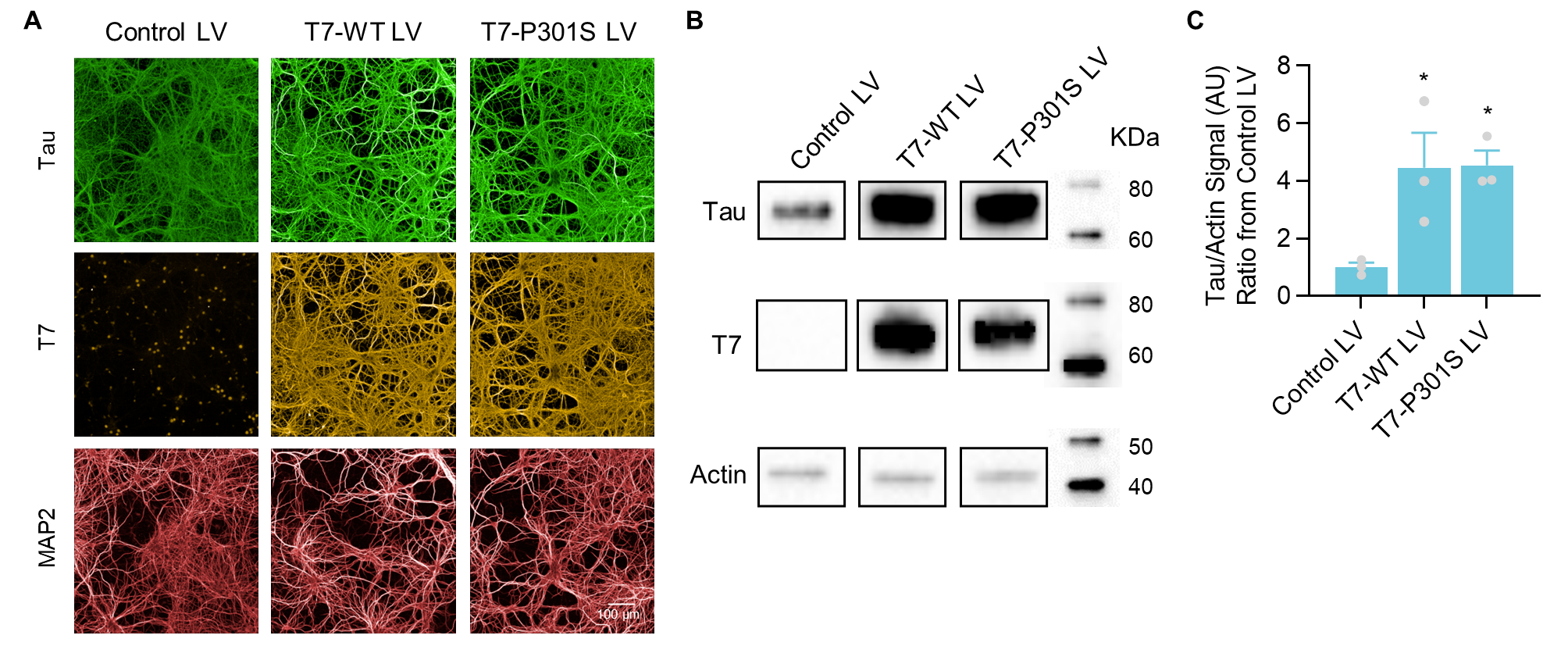

Supplement: S4 Fig — A) Neurons transduced with lentivirus expressing T7-WT, T7-P301S or an empty virus (Control LV) at 5 MOI. LVs were added at DIV4 and cells fixed at DIV18. Examples images of cells fixed and labelled with antibodies against tau (green), T7 (yellow) and MAP2 (red) showing homogenous expression of T7-tau in the whole culture. B) Example of western blot images from lysates from cells treated at DIV6 for 7 days (see S1 Fig for uncropped western blots). C) Quantification of tau levels from B, normalised to beta-actin. Data shown as mean ± SEM, n = 3 independent cultures. One-Way ANOVA with Dunnett’s multiple comparisons test. All conditions tested against Control LV. (TIF) [file pone.0283941.s006.tif]

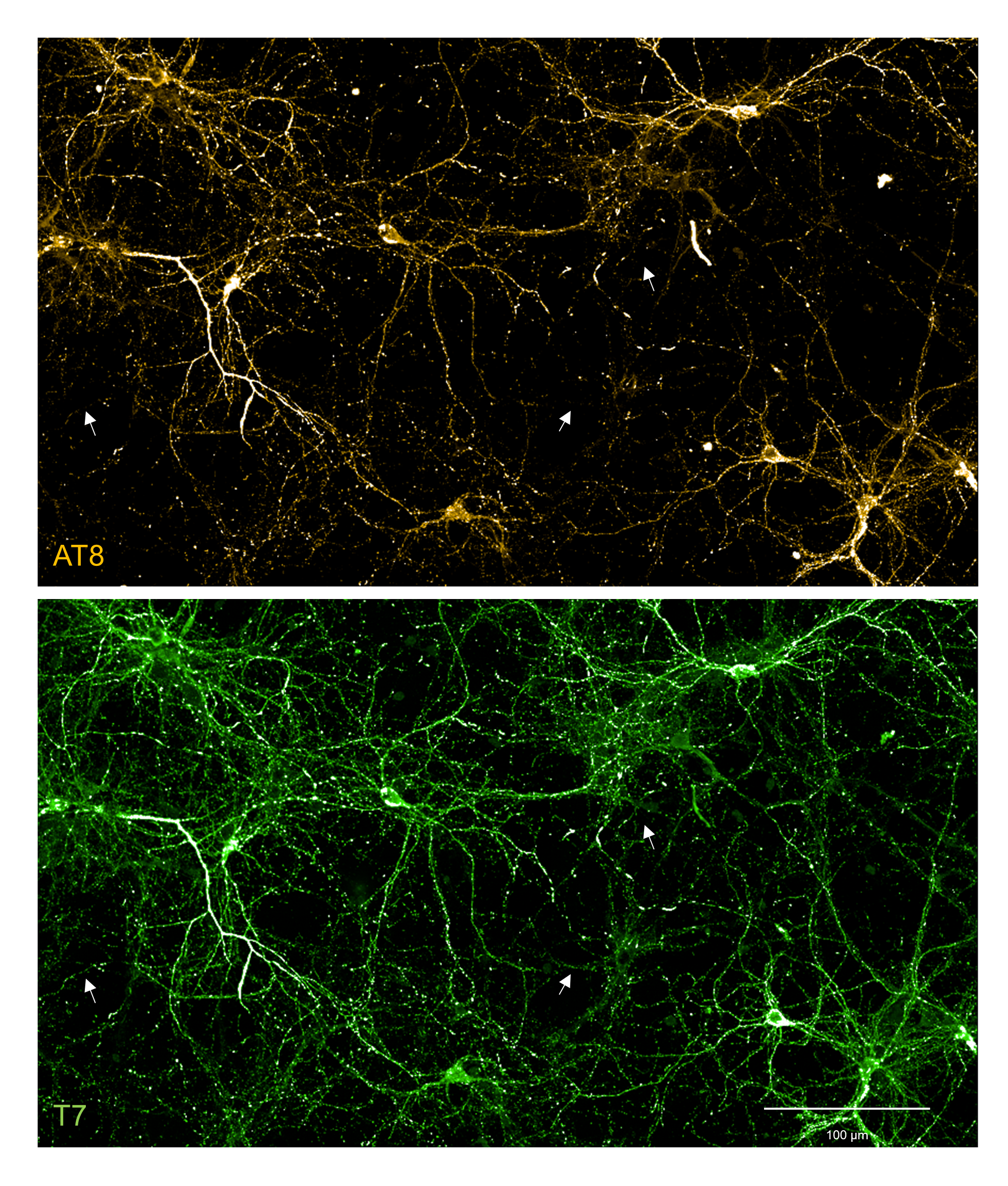

Supplement: S5 Fig — Cells were treated with T7-P301S LV at DIV4, 166 pg/cell of P301S lysates at DIV7, and fixed in the presence of 1% Triton-X 100 at DIV18. Note that AT8 colocalizes with T7 staining, though some T7 immunoreactivity does not show strong AT8 labelling (arrows). (TIF) [file pone.0283941.s007.tif]

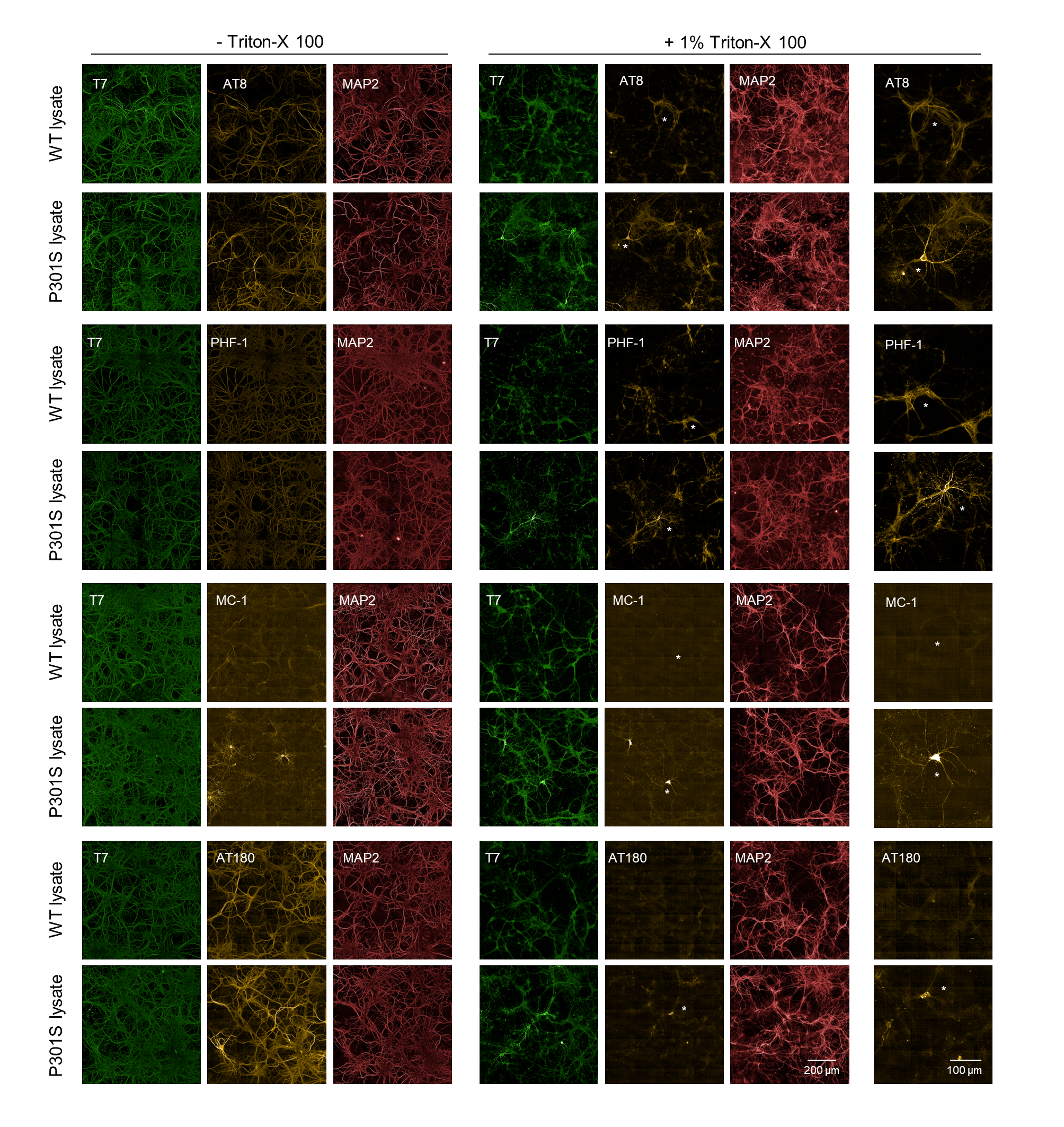

Supplement: S6 Fig — Example images of hTau neurons fixed with 4% PFA in the presence or absence of 1% Triton-X 100, to remove soluble proteins. All cells were treated with T7-P301S lentivirus at DIV4, 166 pg/cell of WT or P301S lysates at DIV7, and fixed at DIV18. IF was performed for T7 (green), MAP2 (red) and tau markers AT8/MC-1/PHF-1/AT180 (yellow). White asterisks provide location information for the zoomed areas on the left. (TIF) [file pone.0283941.s008.tif]

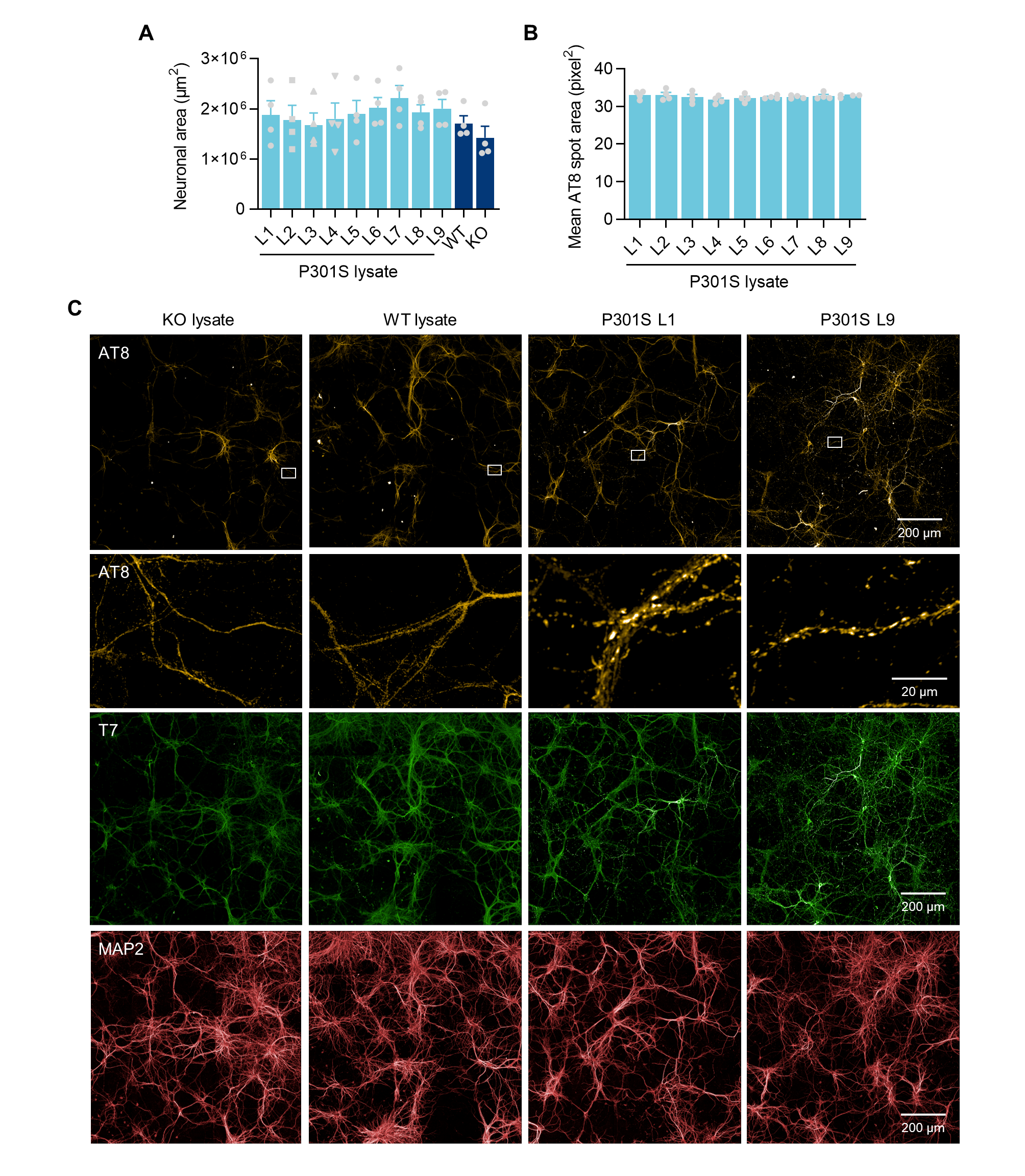

Supplement: S7 Fig — A) Neuronal area (μm2) of hTau neurons transduced at DIV4 with T7-P301S LV and treated at DIV7 with various P301S (9 P301S lysates identified as L1-L9), WT and KO brain lysates. Cells were fixed at DIV18 in the presence of 1% Triton. Data shown as mean ± SEM, n = 4 independent experiments, One-Way ANOVA not significant. B) Mean area of Triton-insoluble AT8 spots (pixel2) from neurons treated as in A. Lysates from different animals induced the formation of inclusions of the same size. Data shown as mean ± SEM, n = 4, One-Way ANOVA not significant. C) Example images of hTau neurons treated as in A. (TIF) [file pone.0283941.s009.tif]
